# Systems approaches in public health: beyond mapping the causes

**DOI:** 10.1186/s12966-025-01766-z

**Published:** 2025-06-12

**Authors:** Loes Crielaard, Mary Nicolaou, Andrew D. Brown, S. Coosje Dijkstra, Fleur ter Ellen, Leonie K. Elsenburg, Angie Luna Pinzon, Wilma E. Waterlander, Karien Stronks

**Affiliations:** 1 Amsterdam UMC, Department Public and Occupational Health, University of Amsterdam, Amsterdam Public Health Research Institute, Van der Boechorststraat 7, Amsterdam, 1081 BT The Netherlands; 2 Faculty of Health, Global Centre for Preventive Health and Nutrition (GLOBE), Institute for Health Transformation, Deakin University, 221 Burwood Highway Burwood VIC 3125, Geelong, VIC Australia; 3 Department of Public Health, Erasmus MC, University Medical Center, Dr. Molewaterplein 40, Rotterdam, 3015 GD The Netherlands; 4 Section of Epidemiology, Department of Public Health, University of Copenhagen, Øster Farimagsgade 5, Copenhagen, DK-1353 Denmark

**Keywords:** Systems science, Systems thinking, System dynamics, Causal loop diagrams, Problem framing, Research question, Problem response, Policy resistance, Unintended consequences, Counterintuitive behaviour

## Abstract

**Background:**

Systems approaches are increasingly adopted in public health, commonly operationalised using system dynamics (SD). In public health, systems approaches have prioritised understanding the current system by describing the causes of a complex problem – e.g. obesity – as a system. It remains challenging to advance from understanding the current system producing undesired outcomes, towards responses to improve outcomes. Rather than creating models of the (entire) system, SD traditionally emphasises specific models to support policy development. While core concepts from SD have effectively been adopted in public health, there may be more to learn from SD when it comes to designing systems approaches that can fulfil the purpose of informing problem responses.

**Methods:**

We reviewed seminal SD literature for clues on how to refine the focus of systems approaches, so that they lead to specific models supporting policy development. We conducted a narrative review, seeking a strategy that can be leveraged in systems approaches in public health. We concentrated on SD’s problem framing strategy, leading to two insights.

**Results:**

Insight 1: Alongside the complex problem at stake (e.g. obesity), consider the intended result of the systems approach (coordination, learning, analysis or transformation). This helps recognise which system components are relevant to problem responses and make methodological decisions accordingly. Insight 2: If investigation of the current system reveals that only radical change can lead to improved outcomes, then proceed to envisioning how the system could be fundamentally transformed to support those desired outcomes. This next step helps to anticipate policy resistance, unintended consequences and counterintuitive behaviour by contemplating how the system would react due to proposed problem responses.

**Conclusions:**

Applying a problem framing strategy, as is commonly done in SD, could make systems approaches in public health better positioned to inform problem responses. Problem framing stimulates the contribution of systems approaches to health policy, prioritising system components relevant to problem responses (Insight 1), which may not be part of the system (Insight 2).

**Supplementary Information:**

The online version contains supplementary material available at 10.1186/s12966-025-01766-z.

## Background

Systems science in public health is receiving growing attention, spurred by the recognition that a novel way of thinking about some persistent public health problems is warranted [1]. The use of systems science stems from the realisation that complex public health problems cannot be addressed with a fixed package of interventions aimed at individual behaviour, isolated socioenvironmental factors or single settings [2]. Public health has traditionally focused on and often resorts to these due to, amongst others, the standardisation and related evaluation of interventions [3]. Complex public health problems, instead, are conceptualised as outcomes of dynamic systems, constituted by interconnected factors operating across various levels, from cells to society [4]. For a complex problem, there is not one unequivocal solution and exploring problem responses can be approached from different angles [5].

While there are other approaches to applying a systems science lens [6–8], in public health, systems thinking is commonly operationalised using system dynamics (SD) [7, 9–11], defined as the application of “informal” models, which are sometimes referred to as (qualitative) maps, as well as “formal” models, which incorporate computer simulation, both with the aim “to uncover and understand endogenous sources of system behaviour” [12]. Core concepts from SD – e.g. boundaries, feedback loops, interconnections, leverage points, nonlinear relationships and paradigms [13] – are central to systems science in public health.

The SD perspective is often implemented in public health through causal loop diagrams (CLDs) [10]. A CLD graphically represents hypothesised causal relationships between factors in a system spanning various levels, focusing on feedback loops [6]. Zooming in on CLDs developed for the complex problem of obesity as an example, our review of 39 papers (see Methods and Additional file) showed that it is common for the models to be geared towards describing the causes/drivers of obesity as a system: describing the current system producing undesired outcomes (16 papers [14–29]) (Box 1). The CLD of the current system is then often used as a basis to search for leverage points, defined as “places [to intervene] in the system where a small change could lead to a large shift in behaviour” [13].

**Box 1:** It is common for causal loop diagrams developed in the context of obesity to be geared towards describing the causes/drivers of obesity as a system: describing the current system producing undesired outcomes

**The main research aim of the causal loop diagram was to map**…
…“factors that could explain the increase in childhood obesity rates in the Netherlands in the past two decades” (Waterlander, Singh et al. 2020)
…”determinants and causes of obesity” (Allender et al. 2015)
…“the underlying system dynamics that drive obesity-related behaviours in 10- to 14-year old adolescents in Amsterdam” (Luna Pinzon et al. 2023)
…“the root causes of childhood obesity” (Nelson et al. 2015)
…“the system driving childhood obesity” (Guariguata et al. 2024)
…”the system of social norms regarding body weight perception and obesity prevalence” (Crielaard et al. 2020)
…“the drivers of adolescent obesity, as perceived by young people themselves” (Savona et al. 2023)
…“the drivers of obesity from adolescents’ perspectives” (Hendricks et al. 2022)
…“the factors contributing to childhood obesity in the community” (Bolton et al. 2022)
…“the underlying logic of obesity drivers for the Campbelltown community” (Maitland et al. 2021)
…“young people’s perceptions of the drivers of adolescent obesity in five European countries” (Savona et al. 2021)
…“the structures, feedbacks and interdependencies among socioculturally specific pathways underlying childhood obesity, in Manhattan’s Chinatown community” (Swierad et al. 2020)
…“the multiple causes of inequities in healthy eating” (Friel et al. 2017)
…“factors [that] explain the unhealthy behaviour (specified for each EBRB [energy-balance related behaviour]) of 10-14-year-old adolescents in Amsterdam (East)” (Emke et al. 2024)
…“the dynamic interactions of the social determinants of NCDs [noncommunicable diseases]” (Sharma et al. 2023)
…“the system drivers of unhealthy food consumption” (Karapici & Cummins, 2024)

Identifying multiple causes and recognising their presence at various levels and involvement in feedback loops is an integral part of systems science and can lead to novel insights regarding complex problems. In addition, in the case of participatory systems modelling, it can lead to outcomes such as consensus, commitment, relationship building and shifts in mental models [30–34]. 

It remains challenging to advance from understanding the current system producing undesired outcomes, towards leverage points and responses to improve outcomes [9, 35]. Even though attempts are made to search for leverage points based on CLDs mapping the current system, the exact procedure for this is only loosely defined. Moreover, we have shown that (seemingly) more structured and transparent approaches that are being applied, such as network analysis, also have issues [36]. Consequently, formulating problem responses is still a challenge in systems approaches (SAs) in public health. A scoping review of CLDs in public health research showed “there was limited discussion regarding how exactly CLDs were to be used to enhance evidence-informed policy and practice” [37].

Yet SD, the field from which the CLD originates, was designed [38] to be “a practical tool policymakers can use to help solve important problems” [39]. Rather than creating models of the (entire) system, SD traditionally emphasises creating specific models to support policy development [39–47]. The aim is to “develop a model to solve a particular problem, not to model the system” [41–43, 48]. While core concepts from SD have effectively been adopted in public health, there may be more to learn from SD when it comes to designing SAs that can fulfil the purpose of informing problem responses [49].

We reviewed seminal SD literature for strategies that can be leveraged when applying SAs in public health. We present our findings by answering the questions: *What strategy does system dynamics offer that can be used to design systems approaches such that they are better positioned to inform problem responses?* and *What can we learn from this strategy to make systems approaches in public health better positioned to inform problem responses?*

## Methods

As the aim of the paper is to generate conceptual insights, rather than a comprehensive overview of the evidence base, we provide a narrative review rather than a systematic or scoping review. First, we reviewed the research aims of CLDs in public health research, to assess what SAs in public health commonly focus on, concentrating specifically on papers describing CLD development in the context of obesity. We take this complex problem as an example, as systems science has often been adopted in this context [37]. We searched Web of Science with search term ‘causal loop diagram obesity’, yielding 39 papers after selection (see Additional file). Our review showed that it is common for CLDs to be geared towards describing the causes/drivers of obesity and their dynamics as a system, i.e. describing the current system producing undesired outcomes (16 papers [14–29]) (Box 1).

Second, we reviewed seminal SD literature for clues on how to refine the focus of SAs, so that they lead to specific models supporting policy development. We conducted a narrative review, seeking a strategy that can be leveraged in SAs in public health. We first revisited *Community Based System Dynamics* by Hovmand [50], given its significance in informing the application of (especially community-based) SAs in public health. We concentrated on the problem framing strategy presented in this book, as it was devised to help refine the focus of SAs so that they can support different purposes – coordination, learning, analysis and transformation [50]. We then examined the background literature for this strategy [51, 52] and cross-checked its theoretical basis with a sample of other seminal SD literature relied on in public health research (22 papers/books [12, 13, 35, 39–48, 53–61]) (see Additional file for quotations of all relevant text considered).

To select a sample of SD literature that is relevant to SAs in public health with the purpose of policy development, we reviewed the reference list of *Systems thinking for noncommunicable disease prevention policy: Guidance to bring systems approaches into practice* by the World Health Organization (WHO) (keywords ‘systems analysis’/’noncommunicable diseases’/’policy-making’/’health policy’/’stakeholder participation’/’capacity-building’). From the WHO reference list (which also includes *Community Based System Dynamics* by Hovmand), we selected those SD authors that have written at least three papers among the most cited in *System Dynamics Review* (the primary SD journal). This makes them key authors in SD: Cavana, Ghaffarzadegan, Homer, Richardson, Saeed, Sterman and Vennix. We reviewed a selection of their most cited key methodological publications (not necessarily published in *System Dynamics Review*), led by keywords related to the problem framing strategy: ‘research question’/‘issue’/‘problem’/‘framing’/‘fram*’/‘purpose’/‘restructur*’/‘structur*’/‘policy’/‘policies’. From authors featured in the WHO reference list, we also reviewed three key methodological publications not covered by the aforementioned selection but especially important to SAs in public health (by Meadows and by Johnston and colleagues [13, 35, 61]).

It was not appropriate or possible to involve patients or the public in the design, or conduct, or reporting, or dissemination plans of our research.

## Results

### What strategy does system dynamics offer that can be used to design systems approaches such that they are better positioned to inform problem responses?

#### The problem framing strategy

**Fig. 1:**
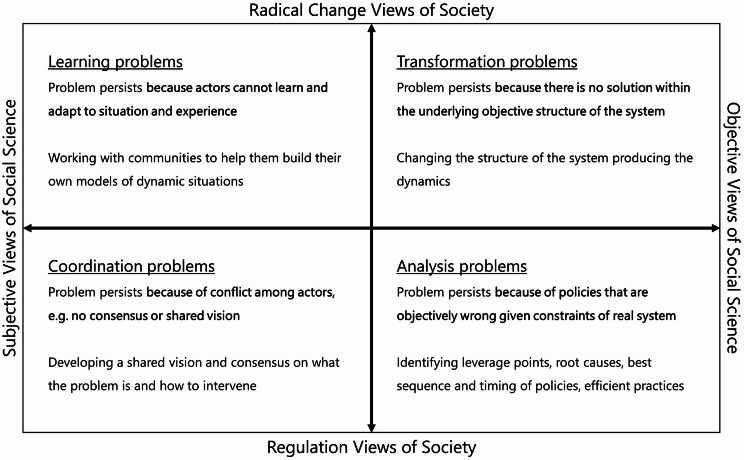
The problem framing matrix, as presented by Hovmand in his book Community Based System Dynamics [50, 62]

The problem framing matrix (Fig. 1) and accompanying strategy, presented by Hovmand in his book *Community Based System Dynamics* [50], can be used to refine an SA’s focus so that it converges to an idea for a problem response. Hovmand’s approach to community-based SD involves building community capacity to respond to complex problems by using methods from SD through a series of SAs over time. In this over time perspective, SAs may serve various purposes, and the purposes of different SAs may evolve. From a capacity building perspective, it is key for community members to recognise when to use SD in what way.

Hovmand suggested to reflect on the type of problem, and related “diagnostic” questions, during early discussions (with community partners, in the case of community-based SD) about shaping an SA, prior to formulating a project description and forming a core modelling team [50]. Related “diagnostic” questions recommended by Hovmand revolve around e.g. whether the problem is dynamic and whether SD is the most suitable method to generate the insights being sought [50]. This implies that understanding the type of problem is considered to be a foundational step in an SA.

The problem framing strategy holds that the choice for how to shape model building processes and how to make methodological decisions, and therefore, the resulting model, depends on the intended result of the SA. There are four purposes of SAs connected to four types of problems (Fig. 1).

**Box 2:** Problem framing by distinguishing between problems of coordination, learning, analysis and transformation, as presented by Hovmand [50, 62]

**Why does the problem persist?**
• The “problem persists *because of conflict among actors*,* e.g. no consensus or shared vision*”: a coordination problem (subjective*regulation).
• The “problem persists *because actors cannot learn and adapt to situation and experience*”: a learning problem (subjective*radical change).
• The “problem persists *because of policies that are objectively wrong given constraints of real system*”: an analysis problem (objective*regulation).
• The “problem persists *because there is no solution within the underlying objective structure of the system*”: a transformation problem (objective*radical change).

The strategy and the matrix imply that, at the outset of the SA, the question ‘Why does the problem persist?’ should be asked [50, 62] (Box 2).

This draws the focus to system components that could explain why previous actions have failed – i.e. the problem *persists* despite former efforts [13, 45] – and are thus relevant to problem responses. Correspondingly, consider this research question suggested for guiding CLD development: “Why does issue X/trend Y occur, despite our efforts to solve the issue/interrupt the trend, while Z is our goal?” [63].

Hovmand stressed the importance of determining the primary barrier to addressing the problem to refine an SA’s focus, which “involves problem framing and will ultimately inform the purpose of a model in a project” [50]. Problem framing concerns “the idea that how problems are defined has a lot to do with the solutions being sought” [50]. The answer to the question ‘Why does the problem persist?’ helps decide on (one of) the (primary) barrier(s) that could explain the problem’s persistence. What is it about the problem that needs to be attended to right now to work towards improved outcomes? What kind of solution are we seeking? Hovmand distinguished between problems of coordination, learning, analysis and transformation, which each confront different barriers [50, 62]. For example, if the barrier is conflict among actors, the aim underlying the SA and a potential model is to achieve coordination. The type of problem relates to why we are applying SD, what we seek to achieve with the SA: “what changes (…) is not the SD method, but what we ask of it, specifically the purpose of a model” [50].

Hovmand ordered the types of problems along two axes. On the vertical axis, Hovmand – building on Burrell and Morgan [52] and Lane [51] – differentiates problems requiring regulation and problems requiring radical change [50]. Regulation problems are framed “in terms of fixing or maintaining the status quo”, where there is “a basic commitment to accepting the structure of the system as it is” [50]. Radical change problems are framed in terms of “rejecting or replacing the status quo”, where there is a basic commitment to “rejecting the current system or seeking to restructure it in some fundamental ways” [50].

On the horizontal axis, Hovmand – again in line with Burrell and Morgan [52] and Lane [51] – separates objective and subjective views of social science. The objective perspective conceptualises “the social world as if it were a hard, external, objective reality”, which “expresses itself most forcefully in a search for universal laws which explain and govern the reality which is being observed” [52]. The subjective perspective emphasises “the importance of the subjective experience of individuals in the creation of the social world”, where “the principle concern is with an understanding of the way in which the individual creates, modifies and interprets the world” [52].

This distinction implies that the contribution of actors taking part in the SA is a function of the intended result of the SA [47, 58]. What can these specific actors contribute to addressing the problem? Why is their contribution needed? For the objective perspective, the contribution of actors in the SA seems to mainly lie in pointing to facts that may have been overlooked otherwise (e.g. because only actors with a specific background are aware of the relevance of a specific factor to the problem). For the subjective perspective, conversely, the contribution of actors seems to mainly lie in offering insight into their own thought processes (e.g. based on what conceptualisation of the problem do actors make policy decisions).

Hovmand explained that the intended result of applying SD could shift over the course of a project, as the perceived primary barrier to addressing the problem may shift, making the project a series of SAs [50]. For example, the initial reason hypothesised for a problem’s persistence may be that the policy in place does not match with the real system. Here, some system components of the current system leading to policy resistance are hypothesised to have been overlooked whilst formulating responses to improve outcomes, and understanding those components is hypothesised to be relevant to problem responses – meaning that you are dealing with an analysis problem. If then improved policy has been implemented that takes account of the previously overlooked components and even with this improved policy, the problem persists, this might mean that it cannot be addressed within the current system. The problem has now become a transformation problem, requiring restructuring of the current system [13, 35, 43, 44, 61].

In practice, the intended results of a project often span multiple areas within the problem framing matrix. Given SD’s focus on change over time, we encourage SAs to be conceptualised as dynamic, and to even consider how multiple projects over time connect to form an overarching ‘SA’ to a collection of complex problems. In this framing, as the project evolves, the purpose may shift between different areas in the problem framing matrix. The point is to identify at any given point in time which part of the matrix may be most relevant, and act accordingly.

While for regulation problems, the aim is unravelling the structure of the current system producing undesired outcomes and assessing how outcomes could be improved while keeping the system as it is (through coordination/analysis) (“improving the current system”, “choosing among a set of known alternatives” [62]), for radical change problems, the aim is envisioning how the system could be restructured to improve outcomes (through learning/transformation) (“looking for the system to evolve into something new” [62]). In addition, while for subjective problems the aim is mainly to support actors in their understanding of the problem (through coordination/learning), placing less emphasis on facts, for objective problems the aim is mainly to explain the aetiology of the problem (through analysis/transformation), placing more emphasis on facts.

#### The system versus the problem in the broader system dynamics literature

The problem framing strategy fits in the broader SD literature, which underlines the importance of problem framing at the outset of an SA [13, 41–43, 47, 48, 53, 54, 58, 60]. In *Best practices in system dynamics modelling*, different model purposes are distinguished, such as strategy development, policy analysis, theory building, education and training [48]. Hovmand's matrix is often taught in introductory SD courses [62, 64], which tend to dedicate a section specifically to problem framing. It builds on Lane’s seminal work on social theory and SD practice [51], which again rests on Burrell and Morgan’s contribution describing how different social theories require the use of different methodologies [52]. The matrix is an implementation of one of SD’s “fundamental principles for good modelling”, namely that “one must always model a problem, never the system” [41–43, 48], where “it [the purpose, i.e. addressing the problem] provides the criteria to decide what can be ignored so that only the essential features necessary to fulfil the purpose are left” [43].

While modelling the problem is a core principle in SD, it could be argued that modelling the system has been the main practice in SAs in public health as they often concentrate on visualising multiple causes and their dynamics in a map. Shifting the focus means that instead of investigating the entire system behind the complex problem, we zoom in on those system components that might explain why previous actions have not managed to address the problem – potentially because of unforeseen endogenous sources of system behaviour [13, 35, 39–41, 43, 45, 54–56, 59, 60]. In this way, more effective problem responses can be proposed [12, 38, 39, 43, 54–56].

That uncovering unforeseen endogenous sources of system behaviour is a main purpose of SD, is explained in the first section of one of SD’s foundational works, Sterman’s *Business Dynamics*, titled *Policy resistance*,* the law of unintended consequences and the counterintuitive behaviour of social systems* [43]. Policy resistance is defined as “the tendency for interventions to be delayed, diluted or defeated by the response of the system to the intervention itself [65]” [43]. Each of these concepts – policy resistance, unintended consequences and counterintuitive behaviour – implies there is an anticipated effect that an action will have on outcomes, but that this expectation is not being met [13, 35, 39–41, 45, 60, 61]. That this expectation is not being met is the motivation for turning to the methods of SD [13, 35, 39–41, 43, 45, 46, 55, 56, 61]: why does the problem persist?

### What can we learn from this strategy to make systems approaches in public health better positioned to inform problem responses?

#### Insight 1: Alongside the complex public health problem at stake – e.g. obesity – it is important to at the outset consider the intended result of the systems approach – coordination, learning, analysis or transformation.

While public health problems of various natures – from obesity, to depression, to COVID-19 [37] – can be framed as complex and studied from a systems perspective, the reasons for doing so may vary. Reasons diverge from developing a shared vision (coordination, e.g. using systems thinking to study mental health as a societal instead of an individual outcome [66]) to identifying best sequence/timing of policies (analysis, e.g. using systems thinking to study the potential unintended consequences of sugar-sweetened beverage taxation [67]). Also for the same complex problem, SAs may be utilised to confront varying barriers to addressing the problem.

The reasons underlying the adoption of an SA determine what specific system components should be explored [13, 41, 42]. This would prevent us from looking at the entire system when this is not necessary. For example, if we want to achieve coordination, we may only have to concentrate our efforts on the level of society and not cells [42, 43]. Correspondingly, the underlying aim dictates all aspects of the methodology [42, 43, 47, 58], such as the emphasis on thought processes or aetiology, and thereby the contribution of actors, the role of data (e.g. the importance of fact-finding) and the means of modelling (e.g. the necessity for exact numbers).

From the outset of the SA, both the complex public health problem (the subject, e.g. obesity) and what we seek to achieve with the SA (the object, e.g. transformation) should be clear (Box 3). This extra layer to problem framing does not tend to be prespecified in literature documenting SAs in public health. It is sometimes assumed that an SA will be able to achieve multiple aims simultaneously, which could result in difficulties in deciding where to focus efforts and making methodological decisions [10, 49].

**Box 3:** Distinction between the complex public health problem/the subject of the systems approach, and the intended result/the object of the systems approach

	Definition
Complex public health problem	The **subject** (defined as “*the thing that is being discussed*,* considered or studied”* (Cambridge Dictionary)) of the systems approach – e.g. obesity. The complex public health problem that persists despite former efforts. *We adopt a systems approach to address complex public health problem X.*
Intended result of the systems approach	The **object** (defined as “*a reason for doing something*,* or the result you wish to achieve by doing it*” (Cambridge Dictionary)) of the systems approach – e.g. transformation. “The most immediate barrier (…) to addressing the problem” (50), the reason for the persistence of the problem, what it is about the problem that needs to be attended to right now to work towards improved outcomes, what we seek to achieve with the systems approach. *We seek to achieve coordination*,* learning*,* analysis or transformation with the systems approach to confront barrier Y*,* which we hypothesise has kept us from addressing complex public health problem X.*

The importance of concentrating an SA on its intended result, and not assuming that it can achieve multiple aims simultaneously, was recognised in the evaluation of the LIKE programme, which employed SD and participatory action research to address childhood obesity in Amsterdam [68]. After five years of LIKE, the research team reflected that the substantial amount of time they spent on understanding how the current system produces undesired outcomes (coordination/analysis), while useful, proved to be in competition with the resources they could allocate to envisioning how to restructure the system to improve health outcomes (learning/transformation), where the latter was LIKE’s primary objective [69]. For example, a significant amount of time was dedicated to creating a CLD covering all factors and causal relationships that influence diet. However, the focus perhaps should have been more on why it remains difficult to make the current food environment healthier, despite all efforts. Based on that, the problem analysis could have been more targeted: this might have shifted the emphasis away from generating a ‘complete’ CLD on the topic of diet in relation to childhood obesity, toward examining, for instance, why schools are not implementing healthy canteens. In the case of LIKE, this would have also better aligned the problem analysis with the strong momentum that existed at that moment in time to develop local policies targeting the food environment, possibly enabling the recognition of leverage points specific to that policy window.

#### How could Insight 1 be applied in practice?

Depending on whether the SA specifically seeks to achieve coordination, learning, analysis or transformation, the framing of the model building processes and related methodological decisions and, therefore, the resulting model, will differ. Prespecifying the type of problem allows concentrating efforts on the relevant system components from the outset [43]. We present examples of SAs to the complex public health problem of obesity (i.e. the subject) that had varying objects (Table 1) [15, 70–72]. The studies mentioned were identified by us as being illustrative of the different types of problems and SAs. Whether they made use of the problem framing strategy, however, was not explicit in the respective publications. The examples illustrate how determining the intended result of the SA can refine the focus, foregoing the need for exhaustively mapping causes, when the mapping process could in fact concentrate on those system components that might explain why previous actions have not managed to address the problem.

Obesity was considered the result of a coordination problem in the literature-based model of Hagenaars and colleagues. The SA intended to expose the feedback loops that explain how stakeholders disagree on the framing of obesity as either an individual problem (biomedical/commercial weight-loss sectors) or a societal problem (communities/non-health government sectors), where the current system was found to reinforce the individual framing [70]. Allender and colleagues approached it as a learning problem and created a model together with community stakeholders, which aimed to “build their [the stakeholders’] own capacity to understand and address complex public health issues like childhood obesity” [15]. Sawyer and colleagues developed a model in answer to obesity as an analysis problem. They systematically synthesised literature through an umbrella review leading to an evidence-based map of the current system, where the SA aspired to “enhance the development and assessment of effective policies and interventions” [71]. The simulation model of Brown and colleagues can act as an example of a model developed for a transformation problem. The SA was meant to anticipate whether improving the taste of tap water in a specific community would actually lead to less sugar-sweetened beverage consumption, as may seem evident at first glance, by – together with community stakeholders – exploring how the intervention could be delayed, diluted or defeated by the system’s response [43, 65]. From an analysis perspective, the community recognised that water taste was a driver of sugar-sweetened beverage consumption. Yet, there are plenty of communities with good-tasting tap water that still struggle with sugar-sweetened beverage consumption. Therefore, the modelling process helped stakeholders consider how to approach improved water taste as a transformation problem, where communities can organise to demand big, structural changes, and multiple components of the system can work together differently to encourage different behaviour. Problem responses accounting for the sources of policy resistance discovered through the SA were then implemented in a real-world pilot test [72].

Even though all models describe factors and causal relationships related to obesity (the SA’s subject), they differed in the type of intended result (the SA’s object) and therefore cover different system components, studied through different model building processes with different related methodological decisions. Even though the aims of the SAs vary, they work towards the same desired outcome: a reduction in the prevalence of obesity. Note that even if in two SAs the same factors and causal relationships are focused on – i.e. the two CLDs look exactly the same – each SA may differ in the purpose of the model. For example, a first SA may have sought to systematically synthesise existing evidence to develop a CLD describing the causes of obesity as a system (analysis problem). A second SA could use the same CLD, with the different aim of illustrating the complexity of obesity to local policymakers, so that they can account for this complexity in their work (learning problem).

**Table 1:** Examples of systems approaches seeking to achieve coordination, learning, analysis and transformation to address the complex public health problem of obesity

Complex public health problem	Intended result of the systems approach	Example	Question	Aim of the systems approach and the model	Findings	Desired outcome towards which the systems approach contributes
Obesity	Coordination	**Why we struggle to make progress in obesity prevention and how we might overcome policy inertia: Lessons from the complexity and political sciences** – (Hagenaars et al. 2024) (stock-and-flow diagram)	What are “feedback loops that hinder policymaking on mitigating obesogenic environments and feedback loops that could trigger and sustain policy change”?	To analyse ”the feedback processes that produce current policy inertia in addressing obesogenic environments, leverage points for change and mechanisms that would sustain change (…) providing a detailed analysis of which obesity prevention policymaking strategies might work best in different stadia of obesity prevention policymaking inertia”.	“Sustained prioritisation of policies targeting obesogenic environments requires shared problem ownership of affected communities and non-health government sectors, by emphasising co-benefits of policies that target obesogenic environments (e.g. ultra-processed food taxation for raising revenue) and solutions that are meaningful for affected communities.”	A reduction in the prevalence of obesity
Obesity	Learning	**A community based systems diagram of obesity causes** – (Allender et al. 2015) (causal loop diagram)	“What kind of consensus and insight can a community gain about the problem of childhood obesity using group model building?”	To include “diverse community stakeholders in group model building in order to build their own capacity to understand and address complex public health issues like childhood obesity”.	“It was possible to generate a pictorial representation of one community’s understanding of the systemic causes of childhood obesity using a causal loop diagram. Participants were able to identify and explain determinants in line with systems thinking, identifying positive and negative feedback loops and time delays. The group model building process and iterations of models produced during the sessions facilitated deeper discussions about the complexity of childhood obesity in Portland and a coordinated response to it that would have been more difficult without a model.”	A reduction in the prevalence of obesity
Obesity	Analysis	**Dynamics of the complex food environment underlying dietary intake in low-income groups: A systems map of associations extracted from a systematic umbrella literature review** – (Sawyer et al. 2021) (causal loop diagram)	What is “the underlying system of environmental factors that drives dietary intake in low-income groups”?	“To systematically synthesise existing evidence in order to identify the system dynamics that sustain and reinforce a food environment that influences dietary intake in low-income groups”.	“In order to reshape system dynamics driving unhealthy food environments, simultaneous, diverse and innovative strategies are needed to facilitate longer-term management of household finances and socially-oriented practices around healthy food production, supply and intake. Ultimately, such strategies must be supported by a system paradigm which prioritises health.”	A reduction in the prevalence of obesity
Obesity	Transformation	**System dynamics modelling to engage community stakeholders in addressing water and sugar-sweetened beverage consumption** – (Brown et al. 2022) (system dynamics model)	How “to increase water consumption and decrease sugar-sweetened beverage consumption in Portland, Victoria, a regional town in Victoria”?	To engage “residents of Portland to test a community-based application of SD [system dynamics] modelling to engage with the complexity of shifting sugar-sweetened beverage consumption to water consumption”.	“The model highlights that simply changing the taste of tap water in isolation would be insufficient to improve community water consumption because other interacting feedback loops would reinforce sugar-sweetened beverage consumption, undermining positive change. Instead, combining improving the taste of water with complementary strategies such as role modelling water consumption and reducing marketing of sugar-sweetened beverages would have greater impact.” “Ultimately, the stakeholders agreed to a pilot test of improving the taste of the water, combining both an improvement of water with a marketing and role modelling initiative.”	A reduction in the prevalence of obesity

#### Insight 2: If analysis of the current system reveals that only radical change can lead to improved outcomes, the next step is to envision how the system could be fundamentally transformed to support those desired outcomes.

In our review described above we found evidence that in SAs in public health, the focus has commonly been on the identification of the problem’s causes/drivers to understand the current system (Box 1). We argue that keeping the spotlight on the current system while searching for leverage points means implicitly assuming we are dealing with a regulation problem requiring coordination/analysis, i.e. we believe that the solution to the problem can be found in the current system. For some problems, we need to move beyond the idea that we will know where to intervene when we have developed a thorough understanding of the current system. Only for a regulation problem can outcomes be improved while keeping the system as it is.

The reasoning - that understanding how the current system produces undesired outcomes is all that is needed to formulate problem responses - could be moulded by the traditional epidemiological perspective. Specifically, if a research question is aimed at identifying the cause of a problem, the answer will logically lead to the solution of the problem – where the solution consists of directly addressing the identified cause [55]. In the classic tale of Modern Epidemiology, John Snow identified contaminated water as the cause of cholera, prompting him to formulate a problem response that would eliminate this cause: removing the handle of the water pump with contaminated water. For an analysis problem such as this one, which typifies much of public health research, understanding the causes and the current system leads to recognising where to intervene: health outcomes can be improved by tweaking a component of the current system [13].

If the public health problem requires radical change, the analysis should go beyond examining the current system and the factors that currently function as causes. The analysis should also envision how the system could be transformed to improve outcomes [13, 35, 43, 44, 57, 61] – from a problem-oriented perspective to a solution-oriented perspective [73]. For example, a focus on understanding the current system can help to discern the paradigm that it operates from, e.g. the maximisation of economic growth [16], but this insight into how the system presently operates does not by itself lead to grasping how it could be restructured to maximise public health instead. In Meadows’ words: “So, how do we change the structure of systems to produce more of what we want and less of that which is undesirable?” [13].

While it is evident that transforming a system – such as the food system, which operates on a global scale – is often unfeasible within the scope of a single SA, acknowledging that transformation is necessary to improve outcomes can still be valuable for guiding next steps. First, it can direct the analysis toward generating evidence that may, in the long term, support policies aimed at system transformation. For instance, transition theory highlights the importance of developing a shared vision and mission – such as defining what a healthy, economically viable food environment looks like [74] – before meaningful change can occur. In this context, it may be more productive for an SA to focus on contributing to such a vision, rather than on producing evidence that supports regulation, looking for the best alternative “among a set of known alternatives” [62] that are already believed to not be sufficiently effective. Second, this acknowledgement can help reframe the problem at a more manageable scale, where transformation is achievable. As illustrated in the LIKE example earlier, recognising the need for systemic change at the global level, while accepting that such change may be beyond reach, can lead to more actionable insights by focusing on what can be restructured locally, rather than expending resources on further analysing elements beyond the project’s influence.

As we understand it, in a learning problem, the primary barrier to addressing the problem being confronted is that *the mental model of the system* needs to be shifted, while in a transformation problem, the primary barrier is that *the physical system* needs to be restructured. The example of Forrester, as reiterated by Meadows, is that the tax system of a country – no matter how it is structured – is a reflection of the mental model that its citizens have of what constitutes a fair tax system [13]. Shifting our mental models can be a prerequisite for restructuring our physical systems. As phrased by Sterman: “As our mental models change, we change the structure of our systems, creating different decision rules and new strategies. The same information, interpreted by a different model, now yields a different decision.” [43, 47, 54, 55].

Leverage points, places within a system where a small shift can lead to significant change, are conceptualised along a continuum from low to high effectiveness, leading to a hierarchy of leverage points. When examining descriptions of higher level leverage points in key SD literature on this topic [13, 35, 61] (Box 4), e.g. Meadows’ Places to Intervene in a System [13, 61] and the Intervention Level Framework [35], through the lens of maintaining the current system versus restructuring it, we see that lower level leverage points typically involve minor adjustments to system structure, such as tweaking parameters, which are deemed to only result in limited system change. Higher level leverage points, on the other hand, target system structure, by shifting mindsets, goals or system rules, and are considered capable of driving more transformative, radical change. Here, radical change can be defined as “an episodic and transformative change pursuit that is fundamentally about shifting the status quo by altering the elemental form and function of a system” [75].

**Box 4:** Examples of higher level leverage points associated with fundamental changes to the system’s underlying structure

**An intervention on system structure** as described in the Intervention Level Framework: “Actions at this level will **shift the system structure by changing system linkages or incorporating novel elements.**” (Johnston et al. 2014)
**An intervention on information flows** – i.e. “the structure of who does and does not have access to information” – as described in Meadows’ Places to Intervene in a System: “It’s not a parameter adjustment not a strengthening or weakening of an existing feedback loop. It’s **a new loop**,** delivering feedback to a place where it wasn’t going before**. (…) Missing information flows is one of the most common causes of system malfunction. Adding or restoring information can be a powerful intervention, usually much easier and cheaper than rebuilding physical infrastructure.” (Meadows, 2008)
**An intervention on self-organisation** – i.e. “the power to add, change or evolve system structure” – as described in Meadows’ Places to Intervene in a System: “Self-organisation means changing any aspect of a system lower on this list–**adding completely new physical structures**, such as brains or wings or computers–**adding new balancing or reinforcing loops**,** or new rules**.” (Meadows, 2008)
**An intervention on paradigms** – i.e. “the shared idea in the minds of society, the great big unstated assumptions, constitute that society’s paradigm, or deepest set of beliefs about how the world works” – as described in Meadows’ Places to Intervene in a System: “ Paradigms are the sources of systems. From them, from **shared social agreements about the nature of reality**, come system goals and information flows, feedbacks, stocks, flows, and everything else about systems. (…) You could say paradigms are harder to change than anything else about a system, and therefore this item should be lowest on the list, not second-to- highest. But there’s nothing physical or expensive or even slow in the process of paradigm change. In a single individual it can happen in a millisecond. All it takes is a click in the mind, a falling of scales from the eyes, **a new way of seeing**.” (Meadows, 2008)

For public health problems requiring radical change, health outcomes cannot be improved while keeping the system as it is. If it is found that the problem cannot be solved within the current system (through coordination/analysis), the next step of considering how to restructure it should be taken [13, 35, 43, 44, 57, 61]. For example, analogous to cholera in John Snow’s time, today PFAS contaminate drinking water affecting public health [76]. While previous research examining the current system has made us aware of the contamination source and the health effects (analysis problem), the primary barrier to addressing the problem has now arguably shifted to there not being a way to reduce PFAS *and* meet commercial interests in the current system (transformation problem) [77]. By understanding the current system we have come to the conclusion that radical change is needed to improve outcomes. The same problem requires us to confront different barriers. This implies that if an SA to address PFAS contamination is started up today, it would make sense to – building on previously acquired information about the current system – invest resources in envisioning how the system could be transformed to improve outcomes, preventing unintended consequences. This is as opposed to starting again from scratch with mapping the current system behind PFAS contamination.

Taking this step, i.e. contemplating how the system would react due to proposed problem responses, could help anticipate policy resistance, unintended consequences and counterintuitive behaviour [13, 35, 39–41, 45, 60, 61]. In the PFAS example, addressing the problem could involve coming up with an idea for a system transformation that both reduces PFAS and aligns with commercial interests. However, the problem response may in turn imply undesired outcomes, which may only become visible through further exploration, e.g. using systems mapping, examining how the system might respond to radical change.

Understanding of policy resistance, unintended consequences and counterintuitive behaviour may especially be limited if the focus is solely on the causes of a complex problem. For example, suppose that ‘energy intake’ has been included as a key factor in a CLD of the causes of obesity. Based on this CLD, a problem response to reduce energy intake might be suggested. However, this approach relies only on mapping the causes of the problem in the current system, without exploring how the problem response itself might introduce new feedback loops and interactions with other system components that had not been considered yet. Imagining how the system might react to proposed problem responses that imply radical change could help anticipate whether they fit with the existing dynamics and whether they generate new, unforeseen (un)intended consequences.

Even for regulation problems, where outcomes can be improved while keeping the system as it is, zeroing in on causes alone could obscure the leverage points that could be intervened on to improve outcomes. Because the emphasis has been on identifying causes, it is customary for SAs in public health to only concentrate on mechanisms (often reinforcing) that can explain how the problem may worsen and not on mechanisms (often balancing [13]), already part of the system, that may alleviate it [16, 78]. This is the result of only causal, and not counteracting, factors being within the scope of the research question. This means that leverage points that are already part of the system that could be intervened on to steer towards improved outcomes could be disregarded in the SA and excluded from the model by design. For example, consider the CLD developed from an analysis perspective informed by the research question “Which dynamics drive chronic stress in a context of adverse socioeconomic conditions?” [78]. As noted in the discussion of this paper, this research question exposes the factors and causal relationships that may drive chronic stress, but excludes those that could prevent it, e.g. social support [78].

#### How could Insight 2 be applied in practice?

An approach illustrating how the shift in focus from understanding the current system to anticipating the consequences of restructuring the system could be operationalised was recently presented by Alvarado and colleagues [79]. It is suggested that to reason about the effect of an intervention it is necessary to first develop a CLD representing the current system and then identify causal relationships between the current system and the intervention [79]. The factors and causal relationships relevant to the intervention are considered distinct here from those that describe the current system. This approach proposes a need for two CLDs, where the second one builds upon the first. The first CLD represents the current system, in line with current practice in which we map “an overview of important systemic elements related to an issue” [79]. In the second CLD, the factors and causal relationships related to the intervention are added to the current system, the end result of which is referred to as an “intervention-focused CLD” [79].

Research questions that form the basis for CLDs could be adjusted so that the resulting model serves to anticipate policy resistance, unintended consequences and counterintuitive behaviour for future problem responses. This research question for model development can serve as inspiration: “If we wanted to run an initiative in Hawke’s Bay to improve food environments for children, what would we need to take into account to make sure it was successful?” [80]. Our review of 39 CLD papers returned more examples of CLDs examining factors and causal relationships relevant to the implementation and health impact of interventions/policies (see Additional file).

Hovmand explained that, when a problem is framed as an analysis problem, “through the analysis, we might then discover why the problem cannot be solved as is and seek to view the problem as a transformation problem” [50]. An example of this notion is offered by Kopainsky in the SD Society Summer School [81]. In the studies presented, SD modelling is utilised to address the problem of ensuring long-term food availability in Zambia whilst minimising environmental costs [82, 83]. An attempt is made to find a way to improve system outcomes while keeping the system as it is, testing two alternative policies – fertiliser subsidies and soil organic matter farming practices. It is then discovered through simulations that, while soil organic matter farming practices may lead to improved long-term food availability, there is always a trade-off between food availability and environmental welfare outcomes (e.g. soil degradation or deforestation) in the current production-oriented system that prioritises efficiency. No solution that achieves both high food availability *and* low environmental costs can be discovered within the current system, suggesting that transformation is required if we are to achieve both objectives. As phrased by Kopainsky: “even if you disperse all the money in the world for fertiliser (…) subsidies, it is still not going to solve your problem”, “there really is no way that the food availability problem can be solved within the current system, it can only be solved if one is willing to accept major trade-offs between food availability and deforestation outcomes” [81–83].

## Conclusions

SAs in public health have prioritised mapping the causes of a complex problem as a system. It remains challenging to advance from understanding the current system producing undesired outcomes, towards responses to improve outcomes. Applying a problem framing strategy, as is commonly done in SD, has the potential to help make SAs in public health better positioned to inform problem responses. We presented two insights regarding how such a strategy can be leveraged in SAs in public health.

Insight 1: Alongside the complex public health problem at stake (e.g. obesity), consider the intended result of the SA (coordination, learning, analysis or transformation). This helps recognise which system components are relevant to problem responses and make methodological decisions accordingly. Insight 2: If investigation of the current system reveals that only radical change can lead to improved outcomes, proceed to envisioning how the system could be fundamentally transformed to support those desired outcomes. This next step helps anticipate policy resistance, unintended consequences and counterintuitive behaviour by contemplating how the system would react due to proposed problem responses.

Problem framing stimulates the contribution of SAs to health policy, prioritising system components relevant to problem responses (Insight 1), which may not be part of the system (Insight 2).

## Electronic supplementary material

Below is the link to the electronic supplementary material.

Supplementary Material 1: (1) Review of research aims of causal loop diagrams in public health research, (2) Review of sample of seminal system dynamics literature relied on in public health research.

## Data Availability

No datasets were generated or analysed during the current study.

## Abbreviations

CLD: Causal loop diagram
SA: Systems approach
SD: System dynamics
WHO: World Health Organization
