# Supplementary material for: Systems approaches in public health: beyond mapping the causes
Source: Int J Behav Nutr Phys Act. 2025 Jun 12;22:74. doi: 10.1186/s12966-025-01766-z (PMC12164216; doi:10.1186/s12966-025-01766-z)
Supplement: Supplementary file 1 — Supplementary Material 1: (1) Review of research aims of causal loop diagrams in public health research, (2) Review of sample of seminal system dynamics literature relied on in public health research. [file 12966_2025_1766_MOESM1_ESM.docx]

**Additional file**

**Table of contents**

1. REVIEW OF RESEARCH AIMS OF CAUSAL LOOP DIAGRAMS IN PUBLIC HEALTH RESEARCH page 1-24
2. REVIEW OF SAMPLE OF SEMINAL SYSTEM DYNAMICS LITERATURE RELIED ON IN PUBLIC HEALTH RESEARCH page 25-61
3. **REVIEW OF RESEARCH AIMS OF CAUSAL LOOP DIAGRAMS IN PUBLIC HEALTH RESEARCH**

We reviewed the research aims of causal loop diagrams in public health research, to assess what systems approaches in public health commonly focus on, concentrating specifically on papers describing causal loop diagram development in the context of obesity. We take this complex problem as an example, as systems science has often been adopted in this context (Baugh Littlejohns, Hill, & Neudorf, 2021). We searched Web of Science with search term ‘causal loop diagram obesity’, yielding 39 papers after selection. Our review showed that it is common for causal loop diagrams to be geared towards describing the causes/drivers of obesity and their dynamics as a system, i.e. describing the current system producing undesired outcomes (16 papers) (see Box 1 in paper).

- Search was last conducted on 1-10-2024.
- Search was conducted using Web of Science (All Fields – Search Term: ‘causal loop diagram obesity’, 59 results).
- Protocols and reviews were excluded.
- Papers describing the development of a causal loop diagram in the context of obesity were included.
- For each paper, Abstract, Introduction and Methods (if there were any ambiguities, also Results) were reviewed to identify the main research aim of the causal loop diagram.
- A distinction was made between:

(1a) causal loop diagrams with the main research aim to map factors and causal relationships contributing to **undesired system outcomes** (e.g. obesity/sedentary behaviour),

(1b) causal loop diagrams with the main research aim to map factors and causal relationships contributing to **desired system outcomes** (e.g. healthy weight/active living),

(2a) causal loop diagrams with the main research aim to map factors and causal relationships contributing to **achieving the desired implementation of an intervention/a policy**  and

(2b) causal loop diagrams with the main research aim to map factors and causal relationships contributing to **achieving** **the desired health impact of an intervention/a policy**.

- Papers were classified based on the descriptions provided in the paper of the factors and causal relationships that the study primarily searched for. Excerpts of the descriptions are included in Additional file Table 1.
- 59 papers were identified via the search, 39 papers were reviewed, yielding 16 papers describing a causal loop diagram with research aim (1a), 4 papers describing a causal loop diagram with research aim (1b), 11 papers describing a causal loop diagram with research aim (2a) and 1 paper describing a causal loop diagram with research aim (2b). 3 papers had research aims (1a), (1b), (2a) and (2b), 1 paper had research aims (1a), (1b), (2a) and 3 papers had research aims (1a) and (1b). When papers had multiple research aims, they either addressed all of them, or it was unclear what aims they precisely addressed.

**Additional file Table 1.**

| **Causal loop diagram paper** | **Research aim of the causal loop diagram (description from paper)** | **Category of research aim of the causal loop diagram** |
| --- | --- | --- |
| 1. Understanding obesity-related behaviors in youth from a systems dynamics perspective: The use of causal loop diagrams – Waterlander (2021) 10.1111/obr.13185 | “The aim of this study is to apply systems dynamics methods to gain insights into the complexity of obesity-related behaviors. We report on how we applied systems dynamics methods and on the extent to which this led to new understandings of the problem and its underlying dynamics.”  “We set up a six-step approach for developing the CLDs [causal loop diagrams]. The central aim was to identify **factors that could explain the increase in childhood obesity rates in the Netherlands in the past two decades**. We further operationalized the research aim by focusing on four types of behaviors that are particularly relevant to childhood obesity and are the focus of the AHWP [Amsterdam Healthy Weight Programme] including dietary behavior, physical activity (PA), sedentary behavior, and sleep, although the evidence on the direct link between the latter two behaviors and obesity is less consistent.^21,22^ Hence, the primary question for a CLD was: “**What factors explain the dynamics in dietary behaviors, physical activity, sedentary behavior, and sleep, in the Netherlands in the past two decades?**”” | **1a – undesired system outcomes** |
| 1. A Community Based Systems Diagram of Obesity Causes – Allender (2015) 10.1371/journal.pone.0129683 | “The process created a causal loop diagram representing community perceptions of **determinants and causes of obesity**.”  “The aim of the first phase was to conduct GMB [group model building] to integrate knowledge and experience of stakeholders from different sectors about **the system drivers of childhood obesity in their community**.”  “Data collection: Session 1—Participants received an overview of the project and were then asked to consider obesity as a dynamic problem and identify as many **variables** as they could **that have affected or been affected by obesity in this community over time**. The community were presented with a graph of changing childhood obesity prevalence over time and asked to create their own graphs of other **changes that have occurred over a similar time period affecting or affected by the trend observed in childhood obesity** (known as graphs over time) [16,18].” | **1a – undesired system outcomes** |
| 1. Systems Thinking and indigenous systems: native contributions to obesity prevention – Heke (2019) 10.1177/1177180118806383 | “The purpose of this article is first to describe how two communities have approached the well-being of their young people in initiatives that are grounded in Mātauranga Māori, using the techniques taken from Systems Thinking. In doing so, we also hope to communicate the value of indigenous approaches more broadly and test the value of Systems Thinking as a “bridge” between two knowledge bases:”  “In Kaikohe, the GMB [group model building] focused on their use of Mātauranga Māori to guide the development of their curriculum and how that contributed to the physical, psychological and spiritual well-being of their students.”  “In Uawa, GMB [group model building] was used to explore two distinct areas. The first was their decision to enter teams in the world Waka Ama (outrigger) championships in Hawaii in 2017 and how that could be used to contribute to a healthy community, rather than it being viewed simply as a sporting event for their top athletes. The second area of focus was their “Kai Atua” project, which was focused on changing people’s understanding of and relationship with food.”  “At the second meeting, an initial framing question was put forward as a starting point for the model development. The question was ***What are the factors that are going to influence people in engaging in better levels of physical activity and improved nutrition?*** The above question reflected a non-indigenous perspective, including the idea, common to research in this area, that physical activity and nutrition are the core factors driving obesity. It was quickly rejected and changed to ***How can Mātauranga Māori influence Waiora?*** […] While such translations are always difficult, a reasonable starting point is to read the focusing question as “**How can Mātauranga Māori [a specific indigenous way of seeing and acting in this world], influence Waiora [physical, psychological and spiritual wellbeing]**.””  “This CLD [causal loop diagram] uses a well-known system structure, commonly referred to as “tragedy of the commons,” in which competition for a scarce and common resource leads to all losing out.” | **1b – desired system outcomes** |
| 1. Understanding the system dynamics of obesity-related behaviours in 10- to 14-year-old adolescents in Amsterdam from a multi-actor perspective – Luna Pinzon (2023) 10.3389/fpubh.2023.1128316 | “To develop an understanding of **the dynamics driving obesity-related behaviours in adolescents**, we conducted systems-based analysis on a causal loop diagram (CLD) created from a multi-actor perspective, including academic researchers, adolescents and local stakeholders.”  “In this paper, we aim to identify and understand **the underlying system dynamics that drive obesity-related behaviours in 10- to 14-year old adolescents in Amsterdam**, by conducting systems-based analysis from a multi-actor perspective.”  “Of note, as our focus was in uncovering the system dynamics, we collected data that accounted for the change over time of factors influencing the four targeted behaviours, rather than a static situation. A central question for the collection of data was therefore: “**What factors explain the dynamics in dietary behaviour, physical activity, sleep, and sedentary behaviour, in 10- to 14- year-old adolescents Amsterdam in the past three decades?**.”” | **1a – undesired system outcomes** |
| 1. Systems Thinking in 49 Communities Related to Healthy Eating, Active Living, and Childhood Obesity – Brennan (2015) 10.1097/PHH.0000000000000248 | “Given the complexity of the obesity epidemic and the communities working to address the epidemic,^9,10^ investigators designed and implemented systems science tools and methods as part of the evaluation of 49 Healthy Kids, Healthy Communities (HKHC) CPs [community partnerships]. This article presents results from the GMB [group model building] techniques used to develop and analyze causal maps for each of the 49 sites.”  “As part of the HKHC [Healthy Kids, Healthy Communities] evaluation aim to conduct a qualitative cross-site process and impact evaluation among all 49 HKHC CPs [community partnerships], evaluators incorporated systems science methods, specifically GMB [group model building], to actively involve a wide range of community representatives (eg, residents, elected officials, government agencies, community-based organizations, businesses) in identifying **trends and underlying feedback systems hypothesized by participants as driving local change in health behaviors and obesity**.”  “The GMB [group model building] sessions for the HKHC [Healthy Kids, Healthy Communities] evaluation were intended to introduce systems thinking at the community level by identifying **the essential parts of the system and how the system influences policy and environmental changes to promote healthy eating and active living and to prevent childhood obesity** through causal mapping of feedback loops for each of the 49 HKHC CPs [community partnerships]. Using an inductive approach, GMB participants identified the essential parts of the system through variables produced during a behavior-over-time graph exercise (see companion article in this supplement1). The purpose of this article is to describe the methods, results, and implications associated with a synthesis of the causal maps, or causal loop diagrams, for each of the 49 HKHC CPs. Specifically, this article addresses the following evaluation questions: 1. What were the most prominent variables in the causal loops diagrams across communities? 2. What were the major feedback structures across communities? 3. What implications from the synthesized HKHC causal loop diagram can be translated to policy makers, practitioners, evaluators, funders, and other community representatives?”  “The GMB [group model building] sessions had 2 main activities designed to gain insight into **groups’ common understanding of the policy, system, and environmental work going on in their community related to healthy eating, active living, and childhood obesity**. The first activity was a 60-minute behavior-over-time graph exercise, in which participants individually created and shared graphs of **things that affect or are affected by policy, system, and environmental changes in their community** using a nominal group technique (ie, all participants described their top-ranked graph, followed by the second-ranked graph, and so on, until all graphs were shared or time ran out). The second activity was a causal loop diagram, or structural elicitation, 60-minute exercise, in which participants collectively shared their perceptions of causal relationships among variables generated from the first exercise to develop a causal loop diagram, or system map, illustrating the community’s theory of change.” | **1a,b – undesired system outcomes, desired system outcomes**  **2a,b – desired implementation of intervention/policy, desired health impact of intervention/policy** |
| 1. Using Group Model Building to Understand Factors That Influence Childhood Obesity in an Urban Environment – Nelson (2015) 10.1097/PHH.0000000000000219 | “The coalition conducted a Group Model Building exercise to better understand **root causes of childhood obesity in its community**.”  “The GMB [group model building] process is fully described elsewhere^17^ but is built around 3 main steps: (1) assembling a team of participants; (2) conducting individual behavior-over-time graphs (BOTG) exercise; and (3) group drawing of the causal loop diagram (structural elicitation) 7 exercise. In this context, participants wanted to understand **the root causes of childhood obesity** to evaluate where MCOPP [The Milwaukee Childhood Obesity Prevention Project] is making an impact.”  “Within this process, both BOTG [behavior-over-time graphs] and the causal loop diagram in steps 2 and 3, respectively, were constructed around the statement, “**Things that affect or are affected by policy, system, and environment changes in this community**.”^19^ In step 2, individual participants drew graphs of how **features of the system that affects childhood obesity** have changed from the past to their current state and describe what may occur in the future. | **1a – undesired system outcomes** |
| 1. A systems thinking framework for understanding rising childhood obesity in the Caribbean – Guariguata (2024) 10.1186/s12961-024-01201-y | “The aim of this study is to develop a systems thinking framework to describe **the common complexities of childhood obesity** in the Caribbean region and identify potential areas of intervention.”  “Group model building (GMB) is a form of systems science. Trained GMB facilitators in Puerto Rico, the US Virgin Islands, Barbados, and Trinidad and Tobago convened a group of multi-disciplinary stakeholders in a series of virtual meetings in 2021 to elaborate a hypothesis of **the system driving childhood obesity** represented by causal loop diagrams (CLD).”  “We present a framework causal loop diagram for the Caribbean that links **the complex modifiable determinants of childhood obesity (social, economic and environmental)** using a stakeholder-driven systems approach. The framework recognizes **the common drivers of the rising obesity epidemic**, some unique to the Caribbean and some with considerable overlap with other parts of the world.”  “Understanding **the determinants of the high and rising burden of childhood obesity in the Caribbean** is a key component to developing successful interventions [17].” | **1a – undesired system outcomes** |
| 1. A Theory of Change for Community-Based Systems Interventions to Prevent Obesity – Brown (2022) 10.1016/j.amepre.2021.10.006 | “This paper aims to use systems science conventions to propose a theory of change for community-based interventions aiming to build capacity and use exemplars from systems science for obesity prevention to describe how this approach works.”  “A dynamic hypothesis was created in workshops conducted in 2020 and 2021 by identifying **variables critical to building community capacity for systems thinking**.”  “The aim of this paper is to present a theory of change for CBIs [community-based interventions] to inform public health practitioners and community stakeholders on how to build community capacity in complex systems science interventions.”  “In 2 sessions, authors (ADB, JW, JH, SA, PF, KAB) with experience in system dynamics and CBI [community-based intervention] research developed graphs over time^26^ to identify and describe **the patterns of change in critical factors that affect the success or failure of CBIs to prevent obesity**.” | **2a – desired implementation of intervention/policy** |
| 1. Social norms and obesity prevalence: From cohort to system dynamics models – Crielaard (2020) 10.1111/obr.13044 | “In this study, we model **the system of social norms regarding body weight perception and obesity prevalence** using SDMs [system dynamics models]. These SDMs are designed to test the hypothesis that as overweight becomes normal, the norm might be counteracting health awareness in shaping individual weight-related behaviour.”  “We present this—as a proof-of-concept of studying feedback loops between individual characteristics and group-level processes using this methodology—with the aim to simulate the effect of three scenarios on group-level BMI [body mass index]. These reflect the question ‘what if' weight-related behaviour were driven (1) only by health awareness, (2) only by norms and (3) by their interaction, i.e. health awareness and norms.” | **1a – undesired system outcomes** |
| 1. System mapping with adolescents: Using group model building to map the complexity of obesity – Savona (2023) 10.1111/obr.13506 | “As part of the CO-CREATE project we conducted GMB [group model building] sessions with young people in six countries to create causal loop diagrams showing **the factors that they believe drive obesity**.”  “To explore **the drivers of adolescent obesity, as perceived by young people themselves**, we conducted GMB [group model building] workshops with young people in the Netherlands, Norway, Poland, Portugal, the United Kingdom, and South Africa.^7^”  “The first stage comprises exercises in which participants build “behavior-over-time graphs” (BOTG) depicting **the dynamic factors that they believe drive the problem, which in this case was obesity**. The “reference mode” for the BOTG provides a succinct description of the focus problem with an emphasis on how it has changed over time; we used the prevalence of adolescent obesity in each country.” | **1a – undesired system outcomes** |
| 1. Understanding the LiveLighter® obesity prevention policy processes: An investigation using political science and systems thinking – Clarke (2020) 10.1016/j.socscimed.2019.112757 | “This study sought to advance obesity prevention policy research and practice by applying theories of the policy process to study decision-making processes involved in the adoption of the contentious LiveLighter® social marketing campaign by the Victorian government in Australia. Through analysis of documents and interviews with policy makers, this qualitative study aimed to gain a better understanding of **the dynamic influences on policy decision-making**. Multiple theories of the policy process were used to elucidate **policy drivers** and Causal Loop Diagramming methods were used to illustrate **the LiveLighter® policy decision-making systems**.”  “This paper aimed to examine **the influences on the decision by the Victorian Government to fund the LiveLighter® campaign**, using multiple theories of the policy process to underpin the analysis. The study also sought to communicate qualitative findings through casual-loop diagram (CLD) methods to help illustrate potential ways to leverage policy change.”  “As part of the second stage, an additional process was used to develop a visual overview of **the multifarious elements that influenced policy decision-making** in this context and the interrelationships between these elements. In order to do this, a CLD [causal loop diagram] was created from the analysed data, based on methods outlined by Kim and Anderson (Kim and Andersen, 2012). This involved analysing the data for either explicitly described or implied causal linkages between **policy influences**.” | **2a – desired implementation of intervention/policy** |
| 1. Adolescents' Perspectives on the Drivers of Obesity Using a Group Model Building Approach: A South African Perspective – Hendricks (2022) 10.3390/ijerph19042160 | “The aim of this study was to explore **the drivers of obesity from adolescents’ perspectives** using a systems approach through group model building in four South African schools.”  “For the purpose of this study, system mapping sessions were implemented using the GMB [group model building] technique to explore adolescents’ perceptions of **the determinants of obesity** in four South African schools.”  “This paper reports on a qualitative research study, including generating CLDs [causal loop diagrams] using group model building and comprehensive notetaking, to depict **factors perceived by participants as affecting the diet choices and physical activity of adolescents and hence obesity**.”  “This study followed a systematic method using GMB [group model building] to derive CLDs [causal loop diagrams] qualitatively representing **the determinants of adolescent obesity**. GMB is a well-recognized method for depicting **the drivers of obesity**, and the complexities it entails, to help guide the development of policy responses [17]. This method is a structured collaborative process designed to guide participants through various stages to generate a causal loop diagram, which depicts **the factors they believe contribute to adolescent obesity**.”  “Participants were led through a series of activities as part of session #1, including: individual and group brainstorming on **factors that affect overweight/obesity**; a ‘graph over time’ exercise to demonstrate how some factors have either increased, decreased, or stayed the same over time i.e., their dynamism; small group sessions for participants to share and prioritize the various factors that they brainstormed; and whole group discussions of the participants’ prioritized factors.”  “The nature of GMB [group model building] is such that the focus is on generating FBLs [feedback loops] that depict **the factors that the young people believe contribute to obesity**. Within the final merged map, the research team identified the FBLs [feedback loops] that illustrate **important drivers of obesity** and are potentially important for intervention.” | **1a – undesired system outcomes** |
| 1. Understanding a successful obesity prevention initiative in children under 5 from a systems perspective – Owen (2018) 10.1371/journal.pone.0195141 | “We report on an approach to apply systems thinking to understand the complexity of a successful obesity prevention intervention in early childhood (children aged up to 5 years) conducted in a regional city in Victoria, Australia.”  “A causal loop diagram (CLD) was developed to represent **system elements related to a successful childhood obesity prevention intervention in early childhood**.”  “The aim of this study was: to develop a causal loop diagram to represent and better understand **the dynamic changes of project implementation over time of a successful community-based obesity prevention intervention in children under 5** and generate from this example a general process that can be applied to other projects in public health.”  “Based on initial analysis of the interviews, project implementation was identified as the dynamic variable of interest for the construction of a causal loop diagram (CLD). The dynamic behaviour of project implementation was described as increasing over time, meaning the various activities of the project were developed and implemented over time until the project successfully achieved its aim (as measured by the impact on child weight status). The goal of building a CLD was to further describe and understand **the feedback loops that led to the success of project implementation**.” | **2a – desired implementation of intervention/policy** |
| 1. Generating change through collective impact and systems science for childhood obesity prevention: The GenR8 Change case study – Bolton (2022) 10.1371/journal.pone.0266654 | “Aim: To describe the first 12 months of a participatory whole-of-community systems approach to creating collective action to tackle childhood obesity, called GenR8 Change, in a local government area of Victoria, Australia.”  “The first two GMB [group model building] sessions included 20 key community leaders where a CLD [causal loop diagram] examining **the factors contributing to childhood obesity in the community** was constructed and refined (22 variables GMB1, 53 variables GMB2).”  “The aim of this paper is to describe the process of a participatory whole-of-community systems approach to collective action for childhood obesity prevention. A case-study, GenR8 Change, from a local government area of Victoria, Australia will be presented. The key community-driven actions proposed as solutions to improve children’s eating and physical activity across the community and areas of action implemented in the first 12 months of the intervention will be presented.”  “The GMB [group model building] process provides facilitators and modellers a precise set of scripts to help community members develop a causal loop diagram (CLD) of the community’s mental model of **the drivers of children’s diet and physical activity behaviours** [37].”  “Ambassadors and community members were subsequently invited to attend a 90 minute GMB [group model building] workshop held at a local cafe where the childhood monitoring data were presented, and the case for preventing obesity in childhood explained. The question framing the workshop was “**what drives children’s health in Southern Grampians**?” The activities in this workshop included graphs over time (participants graphed **factors that affect or are affected by childhood obesity in Southern Grampians**), and connection circles (the content of the graphs over time (now called variables) were built into a connection circle as they were shared by participants.”  “Once the CLD was confirmed by the community to be an accurate representation of **the key influences on childrens’ health in their community**, the participants moved on to a series of action planning/prioritisation tasks.” | **1a – undesired system outcomes** |
| 1. Tracking implementation within a community-led whole of system approach to address childhood overweight and obesity in south west Sydney, Australia – Maitland (2021) 10.1186/s12889-021-11288-5 | “Change4Campbelltown aimed to build capacity among key leaders and the broader community to apply techniques from systems thinking to develop community-led actions that address childhood obesity. Change4Campbelltown comprised development of a stakeholder-informed Causal Loop Diagram (CLD) and locally tailored action plan, formation of key stakeholder and community working groups to prioritise and implement actions, and continuous monitoring of intervention actions.”  “The initiative began with the development of a stakeholder-informed CLD, reflecting **the underlying logic of obesity drivers for the Campbelltown community**, and providing the basis for community-led intervention design. In this paper we present a case study for emerging methods to track implementation of actions, and the strength of stakeholder engagement throughout a whole of system approach to address childhood overweight and obesity.”  “This paper presents analysis and discussion of implementation data collected during the Change4Campbelltown initiative. The intervention aims to build capacity among key leaders and the broader community to apply techniques from systems thinking to develop community-led actions to address childhood obesity. Key components of the intervention include; stakeholder-informed development of CLDs [causal loop diagrams], development of locally-tailored action in response to **the drivers of childhood obesity as described in the CLD**, formation of key stakeholder and community working groups to prioritise and implement actions, and continuous monitoring of intervention actions.”  “The CLD [causal loop diagram] was developed by local leaders and community stakeholders during three locally facilitated community workshops. During the workshops **locally-relevant drivers of childhood obesity** were identified, along with the complex, non-linear relationships between those drivers. The resultant diagram served as a logic model underpinning the design of the set of stakeholder-informed activities that comprised the Change4Campbelltown initiative.” | **1a – undesired system outcomes** |
| 1. Understanding Health Promotion Policy Processes: A Study of the Government Adoption of the Achievement Program in Victoria, Australia – Clarke (2018) 10.3390/ijerph15112393 | “This study investigated the policy process involved in the adoption of the Achievement Program, a settings-based health promotion intervention that was a key pillar of the Healthy Together Victoria obesity prevention initiative. The qualitative study utilised multiple theories of the policy process, as well as Causal Loop Diagramming (CLD) methods, to understand **the policy systems underlying the decision to adopt the Achievement Program**.”  “This study sought to contribute to the obesity prevention policy evidence base by investigating the processes involved in the adoption of the Achievement Program health promotion intervention by the Victorian Government, using multiple theories of the policy process [17]. In doing so, the study sought to answer the questions of how and why [16,18], this initiative was adopted by Victorian policy makers. The aim was to help understand potential ways to drive future obesity prevention policy change in this context. To help bring together the theoretical analysis, the research also sought to utilise Causal Loop Diagramming techniques which can provide a heuristic tool for understandings complex policy systems [19].”  “In order to gain a sense of how the influencing factors relate to each other, a CLD [causal loop diagram] of **the Achievement Program policy process** was developed using the approach outlined by Kim and Anderson [46]. This involved analysing the theoretical analysis text data for either explicitly described or implied causal linkages between **policy influences**.” | **2a – desired implementation of intervention/policy** |
| 1. Identifying the views of adolescents in five European countries on the drivers of obesity using group model building – Savona (2021) 10.1093/eurpub/ckaa251 | “The aim of this research was to use the group model building (GMB) method to identify **young people’s perceptions of the drivers of adolescent obesity in five European countries**, as part of the EU-funded Co-Create project.”  “We used GMB [group model building] with four groups of 16–18-year-olds in schools in each of the five European countries (The Netherlands, Norway, Poland, Portugal and the UK) to create causal loop diagrams (CLDs) representing their perceptions of **the drivers of adolescent obesity**.”  “This article reports findings from an international project called ‘Confronting obesity: Co-creating policy with youth’ (CO-CREATE) which uses a complex systems framework to explore—with young people—**the drivers of adolescent obesity** and potential policy actions, across five European countries. For the segment of the project reported here, we conducted system mapping sessions using the group model building (GMB) technique, to produce system maps, in the form of causal loop diagrams (CLD). We show commonalities across all countries, represented in the merged system map, which expresses qualitatively, the adolescents’ perceptions of **the drivers of obesity** and we focus on the key feedback loops (FBLs) in the map, which indicate potential focal points for change.”  “Working with adolescents across five European countries (The Netherlands, Norway, Poland, Portugal and the UK), we conducted system mapping sessions using a GMB [group model building] approach, to produce qualitative, diagrammatic illustrations of **the perceived drivers of obesity** in the form of CLDs [causal loop diagrams]. We then merged the maps into one and identified salient FBLs [feedback loops].”  “The method used for generating the system maps was GMB [group model building]: a structured, collaborative process designed to guide participants through various stages to generate a CLD [causal loop diagram], which depicts **the factors they believe contribute to adolescent obesity**.^15,16,19^” | **1a – undesired system outcomes** |
| 1. Developing a Socioculturally Nuanced Systems Model of Childhood Obesity in Manhattan's Chinese American Community via Group Model Building – Swierad (2020) 10.1155/2020/4819143 | “The purpose of this study was to develop a qualitative and socioculturally tailored systems model of childhood obesity in the Chinese American community in Manhattan’s Chinatown.”  “GMB [group model building] workshops engendered a causal loop diagram (CLD), the visualization of a complex systems model illustrating **the structures, feedbacks, and interdependencies among socioculturally specific pathways underlying childhood obesity, in Manhattan’s Chinatown community**. Thee analysis of CLD revealed that participants considered **the following factors to influence childhood obesity**: (1) traditional social norms affecting body image, how children are raised, parental pressure to study, and trust in health of traditional foods; (2) grandparents’ responsibility for children; (3) limited time availability of parents at home; and (4) a significant amount of children’s time spent indoors.”  “In this study, we sought to develop a socioculturally nuanced model of childhood obesity in the Chinese American community in Manhattan’s Chinatown in New York City (NYC). The purpose of this paper is to describe the GMB [group model building] method used and to present the main findings based on the insights gained from the CLD [causal loop diagram].”  “Throughout the duration of the workshop, the participants engaged in a series of exercises—outlined by the scripts developed for the workshop—to identify **interconnected factors contributing to childhood obesity among Chinese Americans** (see Table 1 for a summary of key activities).” | **1a – undesired system outcomes** |
| 1. Group Model Building: A Framework for Organizing Healthy Community Program and Policy Initiatives in Columbia, Missouri – Thomas (2015) 10.1097/PHH.0000000000000209 | “This article describes a systems-level evaluation of their work to implement healthy eating and active living policy, system, and environmental changes to support healthier communities for children.”  “In 2011, the partnership participated in a Group Model Building session, consisting of 2 primary activities. First, participants sketched behavior-over-time graphs, illustrating the evolution of **community variables related to child obesity**, such as prevalence of walking to school or the marketing of high-sugar drinks.”  “The resulting causal loop diagram is a visual representation of **the interacting systems**, as perceived by the partnership.”  “The purpose of this article is to describe one specific approach to evaluate the work of the Healthy Community Partnership and its 6 Action Teams. Through the Group Model Building methodology, behavior-over-time graphs and a causal loop diagram were developed to identify and analyze **the essential components of the system influencing policy and environmental changes**.”  “During the first activity, participants individually sketched behavior-over-time graphs, which illustrated the evolution of **a community variable related (or believed to be related) to child obesity**, such as prevalence of walking to school or the marketing of high-sugar drinks.”  “The resulting causal loop diagram (see Figure 1) is a visual representation of **the interacting systems**, as perceived by the partnership.” | **1a,b – undesired system outcomes, desired system outcomes**  **2a – desired implementation of intervention/policy** |
| 1. Understanding the dynamics of obesity prevention policy decision-making using a systems perspective: A case study of Healthy Together Victoria – Clarke (2021) 10.1371/journal.pone.0245535 | “This paper utilised political science theory and systems thinking methods to examine **the dynamics underlying decisions regarding obesity prevention policy adoption within the context of the Australian state government initiative, Healthy Together Victoria (HTV) (2011–2016)**. The aim was to understand **key influences on policy processes**, and to identify potential opportunities to increase the adoption of recommended policies.”  “This paper utilised political science theory and CLD [causal loop diagram] methods to examine **the dynamics underlying decisions regarding obesity prevention policy adoption within the context of the Australian state government initiative, Healthy Together Victoria (HTV)**. The aim was to understand **key influences on policy processes**, and to identify potential opportunities to increase the adoption of recommended obesity prevention policies.”  “A two-stage analysis process was undertaken. In the first stage, data were analysed for each of the selected policy interventions separately. As part of this first stage of analysis, a CLD [causal loop diagram] was generated to describe **the dynamics of the policy process for each intervention**. In the second stage, the six individual CLDs were synthesised to produce a meta-CLD of **the HTV [Healthy Together Victoria] policy system** based on themes that were consistent across the six CLDs.” | **2a – desired implementation of intervention/policy** |
| 1. Healthy Kids, Healthy Cuba: Findings From a Group Model Building Process in the Rural Southwest – Keane (2015) 10.1097/PHH.0000000000000250 | “One method of evaluation was to introduce systems thinking at the community level by identifying **the essential parts of the HKHCuba [Healthy Kids, Healthy Cuba] system and how it influences policy and environmental changes to promote healthy eating and active living as well as to prevent childhood obesity in this unique, triethnic, rural community in New Mexico**.”  “One aspect of HKHC’s [Healthy Kids, Healthy Cuba] evaluation was to introduce systems thinking at the community level by identifying **the essential parts of the system and how it influences policy and environmental change related to healthy eating and active living for prevention of childhood obesity**.”  “Participants were asked to first identify **“things that affect or are affected by policy, systems or environment change in your community” related to “healthy eating, physical activity, and/or obesity”**. Participants then created graphs to identify **perceived influences on healthy eating and active living** (eg, cost of healthy foods) and how those influences (or variables) have changed over time (eg, cost of healthy foods has increased).” | **1a,b – undesired system outcomes, desired system outcomes**  **2a,b – desired implementation of intervention/policy, desired health impact of intervention/policy** |
| 1. Exploring the dynamics of food-related policymaking processes and evidence use in Fiji using systems thinking – Waqa (2017) 10.1186/s12961-017-0240-6 | “This study aims to apply systems thinking to identify **the causes and consequences of poor evidence use in food-related policymaking in selected government ministries in Fiji** and to illicit strategies to strengthen the use of evidence in policymaking.”  “The GMB [group model building] workshops mapped **the process of food-related policymaking and the contribution of scientific and local evidence to the process**, and identified actions to enhance the use of evidence in policymaking.”  “The causal loop diagrams produced by each ministry illustrated **the causes and consequences of insufficient evidence use in developing food policies or precursors of the specific actions**.”  “Drawing on systems thinking, this study aims to identify **the causes and consequences of poor evidence use in food-related policymaking in the health and agriculture ministries in Fiji** and to illicit strategies to strengthen the use of evidence informed by the following research question: Where could evidence levers be applied within the food-related policymaking processes in Fiji?”  “This study used group model building (GMB) and a system dynamics approach [28, 35] to gain insights into the connections between **variables influencing the use of evidence in food-related policymaking in the two selected government ministries in Fiji**.”  “We conducted GMB [group model building] in line with a system dynamics approach [28, 35, 36] to gain insights into the connections between **variables influencing evidence use in food-related policymaking in the two selected ministries**.”  “Participants identified **causes and consequences of poor evidence use in food-related policymaking within their ministry from the inception of the policy problem through to policy implementation** [35, 36].” | **2a – desired implementation of intervention/policy** |
| 1. Using systems science to understand the determinants of inequities in healthy eating – Friel (2017) 10.1371/journal.pone.0188872 | “We report on an approach to developing a system oriented policy actor perspective on **the multiple causes of inequities in healthy eating**.”  “The work of the expert stakeholders generated a comprehensive causal loop diagram of **the determinants of inequity in healthy eating** (the HE2 Diagram).”  “This study sought to organise, from a complex systems perspective, current understandings of **how individual and societal level factors interact to create inequities in healthy eating**. It also sought to identify **policy relevant factors and their dynamic interactions**.”  “In this paper we present the results of research conducted with an expert group of policy actors (policy makers, practitioners and researchers), which is part of a larger study looking at policy development and implementation challenges associated with action on inequities in healthy eating. The larger study seeks to answer the overarching question `What kind of insight can policy actors gain about causes of, and solutions to, inequities in healthy eating using systems science methods?'”  “In this study we use a qualitative soft systems method, based on the principles of system dynamics known as `Collaborative Conceptual Modelling' (CCM), to create a causal loop diagram relating to inequities in healthy eating [41]. The method enabled the integration of the knowledge and experience of key stakeholders/experts from different sectors about **the drivers of inequities in healthy eating** and identify relationships, feedback loops and possible unintended consequences.”  “In the May workshop, participants received an overview of the project and an explanation of system science approaches to the study of complex problems such as inequities in healthy eating. The experts were asked to: i) discuss **the problem of inequities in healthy eating and the various factors/variables that affect it**; and ii) develop their individual `mental models' and combined `pair blended' models of connections between variables, and the nature of the relationships between variables (see Table 2).”  “Participants were required to first independently draw diagrams of **factors that influence inequities in healthy eating**.” | **1a – undesired system outcomes** |
| 1. Applying systems thinking in youth-centred participatory action research for health promotion in an underserved neighbourhood – Emke (2024) 10.3389/fpubh.2024.1272663 | “We therefore combined Intervention Mapping, Participatory Action Research (PAR) and system dynamics in the development, implementation and evaluation of actions contributing to healthy EBRBs [energy balance-related behaviours] together with adolescents.”  “Combining the IM [Intervention Mapping], system dynamics and PAR [Participatory Action Research] approaches could prove critically important for developing interventions that tackle the complex problem of adolescent overweight. In this combination, IM provides a systematic and stepwise framework for developing theory- and evidence-based interventions, whilst system dynamics techniques can provide an understanding of **the complexity of the system that influences adolescents’ EBRBs [energy balance-related behaviours]**.”  “Therefore, this paper describes how we combined IM [Intervention Mapping], PAR [Participatory Action Research] and system dynamics in the development, implementation and evaluation of actions contributing to healthy EBRBs [energy balance-related behaviours] together with adolescents.”  “This step involved conducting a participatory needs assessment, which was subdivided into two steps: (1a) gaining insight into the perspectives of adolescents regarding **factors influencing EBRBs [energy balance-related behaviours] (physical activity, screen use, sleep behaviour and dietary behaviour)**, and (1b) gaining insight into how these perceived factors are connected with one another.”  “In step 1b, the PAR [Participatory Action Research] groups developed CLDs [causal loop diagrams] for each of the EBRBs [energy-balance related behaviours] they focused on based on the results from their peer research. The research question underpinning the development of the CLDs was: **what factors explain the unhealthy behaviour (specified for each EBRB) of 10–14-year-old adolescents in Amsterdam (East)?**” | **1a – undesired system outcomes** |
| 1. Dynamics of the complex food environment underlying dietary intake in low-income groups: a systems map of associations extracted from a systematic umbrella literature review – Sawyer (2021) 10.1186/s12966-021-01164-1 | “We aimed to develop and apply novel causal loop diagramming methods in order to construct an evidence-based map of **the underlying system of environmental factors that drives dietary intake in low-income groups**.”  “A systematic umbrella review was conducted on literature examining **determinants of dietary intake and food environments in low-income youths and adults in high/upper-middle income countries**.”  “Therefore, the objective of this study was to develop and apply causal loop diagramming methods to systematically synthesise existing evidence in order to identify **the system dynamics that sustain and reinforce a food environment that influences dietary intake in low-income groups**. We proposed that the system would be arranged around four dimensions of the food environment: accessibility, availability, affordability and acceptability [8] and consequentially sought evidence of **the factors shaping the food environment at the micro- (individual and social factors), meso- (neighbourhood factors) and macro-level (economic and political factors)**.” | **1a,b – undesired system outcomes, desired system outcomes** |
| 1. Improving Park Space Access for the Healthy Kids, Healthy Communities Partnership in Denver, Colorado – Moreland (2015) 10.1097/PHH.0000000000000234 | “Systems-thinking approaches enhanced the Denver partnership’s work to identify and address **the multiple and complex factors affecting the environment changes implemented to increase active living and healthy eating**.”  “Key members of the Denver HKHC [Healthy Kids, Healthy Communities] coalition were invited to participate in a half-day group model-building workshop to create behavior-over-time graphs and a causal loop diagram. These activities were intended to build on the Denver HKHC partnership’s work by identifying **factors that affect or are affected by policy, system, and environmental changes that influence active living, healthy eating or childhood obesity**.”  “Environments (ie, park space, farms, gardens) developed or renovated should consider identifying and addressing **a range of factors that may influence access and utilization of active living and healthy eating**.”  “Denver’s partnership found the experience highly valuable for identifying **the policy, system, and environment change pathways that lead to increases in active living and healthy food access**. In addition, it highlighted the need to identify and address **the multiple and complex change pathways to ensure the outcomes of environment change, especially with park space, implemented in Denver achieve increased access to active living and healthy eating**.”  “This article explains systems-thinking approaches that were applied specifically to improving park space access in West Denver. Its intent is to familiarize readers with the benefits of using such approaches to improve outcomes of increasing physical activity behavior through access to park space.”  “A behavior-over-time graph is a workshop participant–designed graph identifying **an influence affecting policy system and environmental changes in Denver related to active living, healthy eating and childhood obesity** and illustrates how the influences have changed over time (past, present, and future). A causal loop diagram examines the relationships among the variables from the behavior-over-time graphs. These activities were meant to build on the Denver partnership’s work by identifying **factors that affect or are affected by policy, system, and environmental changes that influence active living, healthy eating or childhood obesity**.”  “Participants identified a range of **things that affect or are affected by policy, system, and environmental changes in Denver related to active living, healthy eating and childhood obesity**.” | **1a,b – undesired system outcomes, desired system outcomes**  **2a,b – desired implementation of intervention/policy, desired health impact of intervention/policy** |
| 1. Applying a systems perspective to understand the mechanisms of the European School Fruit and Vegetable Scheme – Zolfaghari (2022) 10.1093/eurpub/ckac054 | “This study aimed to apply a systems approach to provide an integrated perspective of the mechanisms of the European School Fruit and Vegetable Scheme (the Scheme) to understand better how to increase its long-term impact on children’s fruit and vegetable consumption.”  “This article provides an integrated perspective of **the multiple settings, agents, components and interconnected mechanisms that are at work in this nutrition policy programme to achieve a long-term impact on children’s FV [fruit and vegetables] consumption** and identify ways to enhance their influence.”  “These documents, together with peer-reviewed studies on the Scheme, enabled initial comprehension of it, including **its main structure, success variables and features related to its effectiveness**.”  “Finally, peer-reviewed articles on **the determinants of children’s FV [fruit and vegetables] consumption and school-based FV programmes** were gathered to further develop and strengthen the initial CLD [causal loop diagram] with empirical evidence.” | **2b – desired health impact of intervention/policy** |
| 1. A community-based system dynamics approach suggests solutions for improving healthy food access in a low-income urban environment – Mui (2019) 10.1371/journal.pone.0216985 | “Little is known about the mechanisms through which **neighborhood-level factors (e.g., social support, economic opportunity) relate to suboptimal availability of healthy foods in low-income urban communities**.”  “This process culminated in the development of causal loop diagrams, based on participants’ perspectives, **illustrating the dynamic factors in an urban neighborhood food system**.”  “The purpose of this article is to describe the process and implementation of a group model building workshop with community members to: (1) learn about **the range of factors (e.g., crime, employment, food stocking in stores, etc.) that affect the neighborhood food system and suboptimal healthy food access for residents**; (2) explore different stakeholders’ perspectives on the nature and consequences of suboptimal healthy food access, and to develop common language between these groups to talk about these challenges; and (3) identify potential points of intervention to improve healthy food access.”  “Six scripts were employed, and outputs from each step contributed to the next, until reaching the final output–a causal loop diagram that depicted participants’ perspectives of **the interaction between factors that related to healthy food access in Central West Baltimore** (Table 1). The main objective of Scripts #1–3 was to elicit discussions on **key factors that contribute to and are influenced by neighborhood disorder, the food environment, and food access**. This was initiated around the question, “**What affects access to fresh and affordable food in Central West Baltimore?**”” | **1a,b – undesired system outcomes, desired system outcomes** |
| 1. Building capacity for the use of systems science to support local government public health planning: a case study of the VicHealth Local Government Partnership in Victoria, Australia – O’Halloran (2022) 10.1136/bmjopen-2022-068190 | “Causal loop diagrams (CLD) representing **localised drivers of mental well-being, healthy eating, active living or general health and well-being of children and young people** were developed by community stakeholders.”  “This paper describes the design of a framework to embed systems thinking as a guiding principle for the delivery of municipal prevention of chronic disease in children and young people. The specific approach to systems dynamics is outlined, alongside the processes used to initially build councils’ capacity, and ongoing support mechanisms to guide continued use of the systems thinking methods. Some results reflecting early outcomes from the local communities are provided.”  “Together, council core facilitation teams and workshop participants created a CLD [causal loop diagram] of **the locally relevant drivers of health and well-being of children and/or young people in their community** and determined the highest-priority leverage points for action.”  “The CLDs [causal loop diagrams] highlighted **drivers of childhood health** and the complex, non-linear relationships between those drivers.” | **1b – desired system outcomes** |
| 1. Generating political commitment for ending malnutrition in all its forms: A system dynamics approach for strengthening nutrition actor networks – Baker (2019) 10.1111/obr.12871 | “To inform new thinking and action towards strengthening NAN [nutrition actor network] effectiveness, we use a systems dynamics theoretical approach and literature review to build initial causal loop diagrams (CLDs) of **political commitment and NAN effectiveness** and a qualitative group model building (GMB) method involving an expert workshop to strengthen model validity. First**, a “nutrition commitment system” CLD demonstrates how five interrelated forms of commitment—rhetorical, institutional, operational, embedded, and system‐wide—can dynamically reinforce or diminish one another over time**. Second, we present CLDs demonstrating **factors shaping NAN effectiveness organized into three categories: actor features, resources, and capacities; framing strategies, evidence, and norms; and institutional, political, and societal contexts**.”  “In this paper, we use a systems approach to generate several CLDs [causal loop diagrams] as **logic models for better understanding the complexity and dynamic nature of political commitment and NAN [nutrition actor network] effectiveness**. The first model, which we refer to as **the “nutrition commitment system,” demonstrates five forms of commitment and several key reinforcing feedback loops (commitment cycles) that connect them**. We then present several further CLDs that demonstrate **the factors that shape NAN effectiveness as an essential driver of nutrition commitment systems**. In doing so, we demonstrate a novel application of systems thinking for informing actions to strengthen NANs and generate political commitment for nutrition during the UN [United Nations] Decade of Action on Nutrition.”  “For this paper, our first CLD [causal loop diagram] was developed to hypothesize how political commitment to nutrition may grow exponentially over the time period of the UN [United Nations] Decade of Action on Nutrition. The second set of CLDs were developed to hypothesize how NANs [nutrition actor networks] could be strengthened exponentially over the same time period.” | **2a – desired implementation of intervention/policy** |
| 1. Building a Prevention System: Infrastructure to Strengthen Health Promotion Outcomes – Bensberg (2021) 10.3390/ijerph18041618 | “This paper reports on findings of 31 semi-structured interviews about participants’ understanding of systems thinking and their reflections of the strengths and weaknesses of the HTV [Healthy Together Victoria] prevention system. A chronic disease prevention framework informed the coding that was used to create a causal loop diagram and a core feedback loop to illustrate the results.”  “There is limited research about the use of systems sciences pertaining to the macro environment in public health [14,15]. This paper uniquely describes the establishment of Healthy Together Victoria’s (HTV) infrastructure for a systems approach to prevention [16].”  “Based on a systematic review, an interpretative framework by Baugh Littlejohns and Wilson (2019), ‘Strengthening systems for chronic disease prevention’, informed the coding [51]. It identified **the parts needed for an effective chronic disease prevention system**.”  “A causal loop diagram (CLD) and a core feedback loop (produced in Vensim^TM^ software) summarize the findings, using Kim and Andersen’s (2012) coding method for system dynamics to generate maps using qualitative text [54]. This involved analyzing the themes for explicit or implied **parts of the prevention system** and the direction of the causal linkages between them to develop the CLD and core feedback loop [23].” | **2a – desired implementation of intervention/policy** |
| 1. Dietary practices, physical activity and social determinants of non-communicable diseases in Nepal: A systemic analysis – Sharma (2023) 10.1371/journal.pone.0281355 | “The objective of this paper was to describe the role of dietary practices and physical activity in the interaction of the social determinants of NCDs [non-communicable diseases] in Nepal, a developing economy.”  “Thematic analysis of the qualitative data was performed, and a causal loop diagram was built to illustrate **the dynamic interactions of the social determinants of NCDs [non-communicable diseases]** based on the themes.”  “This paper assesses the role of dietary practices and physical activity in the interaction of the social determinants of NCDs [non-communicable diseases] in Nepal.”  “A systems map or causal Loop Diagram (CLD) was developed to illustrate **the association of the social determinants of NCDs [non-communicable diseases]** indicated by thematic analysis.” | **1a – undesired system outcomes** |
| 1. A participatory approach to model the neighbourhood food environment – Karapici (2024) 10.1371/journal.pone.0292700 | “In this study, we describe a participatory approach for understanding **the system drivers of unhealthy food consumption**. System dynamics (SD) was used to identify, understand, and visualise **the elements of the neighbourhood food retail system that influence individuals’ eating behaviour**. Group Model Building (GMB), undertaken online with stakeholders (n = 11), was used to funnel existing knowledge and evidence on urban food environments and to build a conceptual system map of **the local food retail environment inclusive of the drivers that influence the decision to purchase and consume meals that are high in fat, salt, and sugar (HFSS), and calories**.”  “The GMB [group model building] generated a comprehensive causal loop diagram (CLD) of **the retail environment inclusive of the drivers that influence the decision to purchase and consume unhealthy meals**.”  “The conceptual model illustrates **the complexity of the factors responsible for inequalities in unhealthy eating**.”  “This paper presents the findings from the first stage of this project. Group Model Building was utilized to gather information on the neighbourhood food retail environment and identify **the key variables that impact the purchasing and consumption of high-fat, high-sugar foods consumed outside of the home**. A Causal Loop Diagram **of the neighbourhood food retail environment** was then created.”  “Group Model Building was used to create a system map of **the neighbourhood food environment and its influence on eating behaviour**.”  “The goal was to create a basic conceptual model that could accurately depict **the important factors related to the local food retail environment**.” | **1a – undesired system outcomes** |
| 1. Using System Dynamics to Understand Transnational Corporate Power in Diet-Related Non-communicable Disease Prevention Policy-Making: A Case Study of South Africa – Milsom (2023) 10.34172/ijhpm.2023.7641 | “This study uses qualitative system dynamics methods to map **the political economy of diet-related NCD [non-communicable disease] (DR-NCD) prevention policy-making** aiming to better understand the policy inertia observed in this area globally.”  “We developed individual then combined casual loop diagrams to generate **a shared model representing the DR-NCD [diet-related non-communicable disease] prevention policy-making system**.”  “Further utilizing systems thinking approaches in this area, the aim of this work was to deepen understanding of the causal complexity of DR-NCD [diet-related non-communicable disease] policy inaction due to multiple inter-dependent political economy mechanisms and different forms of transnational corporate power. Using South Africa as a case study, we apply system dynamics methods to develop several dynamic hypotheses to describe the problem of DR-NCD policy inaction.”  “This study applied a participatory system dynamics modelling method using key stakeholder interviews to iteratively develop several initial causal loop diagrams (CLDs) hypothesizing how, over the past two decades of trade and investment liberalization, transnational corporate power may operate to weaken DR-NCD [diet-related non-communicable disease] prevention policy norms in South Africa.”  “The conceptual model represents **interactions between elements (eg, actions, conditions, and resources) that may explain observed limitations on DR-NCD [diet-related non-communicable disease] policy progress over time despite increasing obesity and NCD [non-communicable disease] prevalence in South Africa**.” | **2a – desired implementation of intervention/policy** |
| 1. A systems framework for implementing healthy food retail in grocery settings – Zorbas (2024) 10.1186/s12889-023-17075-8 | “The aim of this study was to enhance our knowledge of the leverage points for implementing HFR [healthy food retail] initiatives in grocery settings. Our objective was to test the applicability of the START [Systems Thinking Approach to Retail Transformation] map to describe **factors influencing the adoption, implementation and maintenance of several healthy food marketing and promotion initiatives that formed a HFR intervention in a grocery setting**.”  “This study involved a qualitative approach with system dynamics as the main theoretical framework to understand **the mechanisms that drive and hinder HFR [healthy food retail] in grocery settings** [31].” | **2a – desired implementation of intervention/policy** |
| 1. Food system dynamics structuring nutrition equity in racialized urban neighborhoods – Freedman (2022) 10.1093/ajcn/nqab380 | “Research is needed to illuminate **the dynamics structuring food systems in racialized neighborhoods** to inform intervention development.”  “To conduct participatory research examining **the complexity and inequity of food systems in historically redlined neighborhoods** to identify feedback mechanisms to leverage in efforts to transform system outcomes for racial equity.”  “Our objectives here are to: 1) describe a deliberative and situated systems research approach we used to generate insights about **dynamic complexity structuring food systems in historically redlined urban neighborhoods**; 2) characterize an emergent construct, which we labeled nutrition equity, as an aspirational goal of food systems in these neighborhoods; and 3) present causal loop diagrams (CLDs) that depict **underlying feedback mechanisms structuring nutrition equity in racialized urban neighborhoods**.”  “Our aim in conducting this research was to apply a systems lens to examine **complexity and inequity of food systems in historically redlined neighborhoods** with the goal of identifying points of leverage for transforming outcomes of the system to advance racial equity.”  “Building on existing “scripts” developed to support participatory system dynamic modeling (61), 20 interactive workshops were conducted with the core modeling team from 2018 to 2021 with the goal of defining **the preferred state of food systems in racialized urban neighborhoods**, system boundaries for this analysis, and hypothesized causal relations and feedback mechanisms.” | **1a,b – undesired system outcomes, desired system outcomes** |
| 1. Systems thinking for local food environments: a participatory approach identifying leverage points and actions for healthy and sustainable transformations – Wopereis (2024) 10.1186/s12961-024-01199-3 | “Using a participatory approach with local stakeholders, this study aims to gain insight into **the factors and mechanisms underlying the local food environment** and to identify leverage points and system-based actions to foster healthy and sustainable local food environments.”  “During the first workshop (June 2022), **factors and mechanisms influencing the local food environment** were identified and visualized through a causal loop diagram (CLD).”  “The CLD [causal loop diagram] visualises **the factors and mechanisms influencing the local food environment** from the point of view of the community stakeholders.”  “Therefore, using a GMB [group model building] approach with local stakeholders, this study aims to gain insight into **the perceived factors and mechanisms underlying the local food environment** and to identify potential leverage points and system-based actions for healthy and sustainable local food environments.”  “The first GMB [group model building] workshop aimed to gain insight into **the factors and mechanisms underlying the healthiness and sustainability of the local food environment** using existing GMB exercises (Table 1).”  “Based on the outcomes from the GMB [group model building] workshops, insights from the external member-check meeting, and evaluations of the research team, a CLD [causal loop diagram] visualizing **the factors and mechanisms underlying the local food environment** from the point of view of the local stakeholders was developed (Table 1).”  “Figure 1 shows the final CLD [causal loop diagram] illustrating **46 factors and mechanisms that were perceived to shape a healthy and sustainable local food environment**.” | **1b – desired system outcomes** |
| 1. A systems thinking approach to understanding youth active recreation – Koorts (2022) 10.1186/s12966-022-01292-2 | “The aims of this study were: (i) to use systems analysis methods to understand youth active recreation in Victoria, Australia, (ii) identify potential system leverage points to enhance active recreation, and (iii) explore stakeholder views of systems analysis methods for informing practice and policy decision-making.”  “Phase 1: Umbrella review of systematic reviews (2013–2018), synthesising evidence for **correlates, determinants and intervention evidence for promoting active recreation**. Phase 2: Development of three systems models (ActorMap and two ActivMaps), depicting active recreation actors/organisations, correlates, determinants and intervention evidence. Phase 3: Development of causal loop diagrams (CLDs) and identification of leverage points based on the Action Scales Model. Phase 4: Model feedback via stakeholder interviews (n = 23; 16 organisations).”  “The aims of this study were threefold. Firstly, to use systems analysis methods to understand **the active recreation system for pre-schoolers, children and adolescents (herein referred to as children and adolescents) in Victoria**, Australia. Secondly, to identify potential system leverage points using the Action Scales Model [43], and thirdly, to explore stakeholder views of these systems analysis methods as tools to inform practice and policy decision-making.”  “The CLDs [causal loop diagrams] depict **the variables influencing active recreation** that were identified from literature searches (Phase 1), colour coded in the CLD based on the level of influence (Phase 2).” | **1b – desired system outcomes** |
| 1. Mechanisms of scaling up: combining a realist perspective and systems analysis to understand successfully scaled interventions – Koorts (2021) 10.1186/s12966-021-01103-0 | “This paper aims to understand how different contexts of scaling up interventions affect mechanisms to produce intended and unintended scale up outcomes.”  “The purpose of the present study was to combine realist principles and systems analysis (causal loop diagrams to pictorially demonstrate the relationships between contexts, mechanisms and outcomes) to explore **the drivers underpinning implementation outcomes at scale** and how these differed by key academics, practitioners and policymakers involved. Instead of focussing on ‘what went on’ during scale up (i.e., barriers and facilitators experienced) and whether scale up led to a sustainable population health impact; a core aim was to ascertain whether generalisable CMOs [‘Context-Mechanism-Outcome’] existed, which could be applied to a process (scaling up) as opposed to primarily a program. Using CMO configurations, we hypothesise how scaling up processes occur in order to generalise about the mechanisms underpinning scale up.”  “For the purposes of this study, systems analysis was used to understand the relationships within individual CMOs [‘Context-Mechanism-Outcome’] underpinning the scale up process. In this paper, causal loop diagrams (herein referred to as ‘systems models’) provide **a visualisation of the cross-case CMOs** that were identified through interviews.” | **2a – desired implementation of intervention/policy** |

1. **REVIEW OF SAMPLE OF SEMINAL SYSTEM DYNAMICS LITERATURE RELIED ON IN PUBLIC HEALTH RESEARCH**

We reviewed seminal system dynamics literature for clues on how to refine the focus of systems approaches, so that they lead to specific models supporting policy development. We conducted a narrative review, seeking a strategy that can be leveraged in systems approaches in public health. We first revisited *Community Based System Dynamics* by Hovmand (Hovmand, 2014), given its significance in informing the application of (especially community-based) systems approaches in public health. We concentrated on the problem framing strategy presented in this book, as it was devised to help refine the focus of systems approaches so that they can support different purposes – coordination, learning, analysis and transformation (Hovmand, 2014). We then examined the background literature for this strategy (Burrell & Morgan, 1979; Lane, 1999) and cross-checked its theoretical basis with a sample of other seminal system dynamics literature relied on in public health research (22 papers/books).

To select a sample of system dynamics literature that is relevant to systems approaches in public health with the purpose of policy development, we reviewed the reference list of *Systems thinking for noncommunicable disease prevention policy: Guidance to bring systems approaches into practice* by the World Health Organization (WHO) (keywords ‘systems analysis’/’noncommunicable diseases’/’policy-making’/’health policy’/’stakeholder participation’/’capacity-building’). From the WHO reference list (which also includes *Community Based System Dynamics* by Hovmand), we selected those system dynamics authors that have written at least three papers among the most cited in *System Dynamics Review* (the primary system dynamics journal). This makes them key authors in system dynamics: Cavana, Ghaffarzadegan, Homer, Richardson, Saeed, Sterman and Vennix. We reviewed a selection of their most cited key methodological publications (not necessarily published in *System Dynamics Review*), led by keywords related to the problem framing strategy: ‘research question’/’issue’/’problem’/’framing’/’fram*’/’purpose’/’restructur*’/’structur*’/’policy’/’policies’. From authors featured in the WHO reference list, we also reviewed three key methodological publications not covered by the aforementioned selection but especially important to systems approaches in public health (by Meadows and by Johnston and colleagues) (Johnston, Matteson, & Finegood, 2014; Meadows, 1999, 2008).

- Search was last conducted on 22-8-2024.
- Search was conducted using Google Scholar (English language only, Advanced search, Find articles with **all** of the words: system dynamics, Return articles authored by: George Richardson/John Sterman/…).
- Four books that had been selected could not be accessed for review, as shown below.

**Additional file Table 2.**

| **Key author in system dynamics** | **Selected key methodological publications (cited by at least 100) of key author (first author or co-author)** | **Type** | **Relevant text** |
| --- | --- | --- | --- |
| George P Richardson | 1. Reflections on the foundations of system dynamics – Richardson (2011) 10.1002/sdr.462 | Paper | *No relevant text.* |
|  | 2. Best practices in system dynamics modeling – Martinez‐Moyano (2013) 10.1002/sdr.1495 | Paper | “Table 4. Best-practice statements in problem identification and definition  Importance – Highest  Summarized statements – Elicit and thoroughly understand the client's identified problem, Clarify the purpose of the work”  “Table 8. Best-practice statements in model use, implementation, and dissemination  Importance – Average  Summarized statements – Focus on implementation from the start, and target implementable policy options”  “Table 10. Summary of best-practice statements with highest importance and high agreement  In exemplary problem identification and definition, you should …  Speak with and listen carefully and reflectively to problem owners (clients) to identify and understand the problem  Explicitly clarify and state the purpose of the modeling effort (e.g., strategy development, policy analysis, theory building, education, training)”  “Table 11. Best-practice statements with highest importance and low agreement  Problem identification and definition  In exemplary problem identification and definition, two competing approaches emerged: model the system (class) vs. model the problem (case),  Because in this stage you should … Model the class to which the case belongs, not the case at hand OR Model the particular case being studied and identify the class of system to which it belongs”  “In problem identification and definition, there is high agreement regarding the importance of (1) involving problem owners in the modeling process, (2) clearly identifying the purpose of the modeling, (3) formulating a dynamic hypothesis, and (4) clearly articulating the dynamics of the problem using current and expected patterns of behavior.”  “However, there seems to be a lower level of agreement regarding where the focus of the modeling effort should be; i.e., whether to model the system (class of case being studied) or the problem (case). This lower agreement was also captured by Meadows et al. (1982, p. 270) as a tension among global modelers about whether to answer well-defined questions (case) versus representing many aspects of a system (class). In the case of the group of experts in this study, the lower level of agreement regarding model focus may be related to the experts' different backgrounds and professional orientations. Academics tend to favor drawing generic lessons from their modeling efforts (Forrester, 1963, 1973), while practitioners are more interested in the specifics of the cases they study (e.g. Lyneis et al., 2001; Graham and Ariza, 2003).” |
|  | 3. How small system dynamics models can help the public policy process – Ghaffarzadegan (2010) 10.1002/sdr.442 (*same as below Navid Ghaffarzadegan*) | Paper | “Public policies often fail to achieve their intended result because of the complexity of both the environment and the policy-making process. In this article, we review the benefits of using small system dynamics models to address public policy questions. First we discuss the main difficulties inherent in the public policymaking process. Then, we discuss how small system dynamics models can address policy-making difficulties by examining two promising examples: the first in the domain of urban planning and the second in the domain of social welfare. These examples show how small models can yield accessible, insightful lessons for policy making stemming from the endogenous and aggregate perspective of system dynamics modeling and simulation.”    “Despite the high applicability to public policy problems, system dynamics is currently not utilized to its full potential in government policy making. The 2008 system dynamics publications database lists only 94 entries containing the phrase “public policy” out of more than 8800 total entries (System Dynamics Society, 2009).”  “To show how small system dynamics models can be useful for policy making, in this paper we first review five characteristics of public policy problems that make resolution difficult using traditional approaches. These characteristics are policy resistance, the need for and cost of experimentation, the need to achieve consensus between diverse stakeholders, overconfidence, and the need to have an endogenous perspective.”  “Despite addressing diverse policy questions, these models have several common characteristics that illustrate the usefulness of small system dynamics models for policy making more generally. Most notably, both models reveal counterintuitive behavior that is not readily apparent in the absence of an endogenous and aggregate simulation approach.”  “Policy resistance from the environment – The first characteristic of public policy problems is the complexity of the environment in which problems arise and in which policies are made. Such complexity leaves policies highly vulnerable to “policy resistance” (Forrester, 1971b; Sterman, 2000). Policy resistance occurs when policy actions trigger feedback from the environment that undermines the policy and at times even exacerbates the original problem. Policy resistance is common in complex systems characterized by many feedback loops with long delays between policy action and result. In such systems, learning is difficult and actors may continually fail to appreciate the full complexity of the systems that they are attempting to influence. Often, the most intuitive policies bring immediate benefits, only to see those benefits undermined gradually through policy resistance (e.g., Repenning and Sterman, 2002). As Forrester (1971b) notes, because of policy resistance, systems are often insensitive to the most intuitive policies. Policy resistance often arises through the balancing feedback loops that numerously exist in social systems. For example, if a policy increases the standard of living in an urban area, more people will migrate to the area (a balancing loop), consuming resources (e.g., food, houses, businesses), thereby causing the standard of living to decline and reversing the effects of the original policy (Forrester, 1971a). Similarly, when police forces are deployed to control an illegal drug market, drug supply decreases, leading to higher drug prices, more profit per sale, and greater attractiveness of drug dealing. The number of dealers increases, undermining the original policy (Richardson, 1983b). Many more examples exist. These examples illustrate how attempts to intervene in complex systems often fail when policymakers fail to account for important sources of compensating feedback from the environment. Traditional tools that lack a feedback approach may therefore fail to anticipate the best policy actions.”  “We argue that an understanding of the main feedback structure of a system, as provided by a small system dynamics model, is essential to effective policy design. Here, we illustrate the importance of a feedback view to urban policy making through the example of a common policy response to urban decay that has failed in the past. Why do policymakers choose policies that fail? Using the method of partial model testing (Morecroft, 1983; Sterman, 2000), we show that this policy response is in fact intendedly rational for decision makers who fail to account for the feedback structure of the system. Only when the full feedback structure is considered is the likely ineffectiveness of the policy revealed. Thus, by building intuition regarding how feedback affects system behavior, small system dynamics models have a crucial role to play in policymaking.”  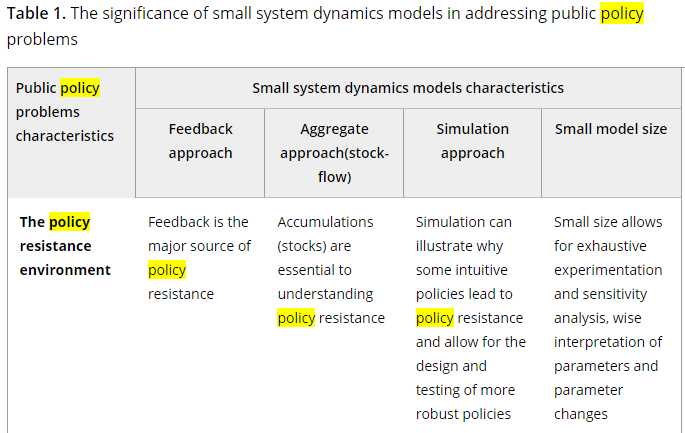  “Such counterintuitive behavior is often an example of policy resistance. Too often, policies fail due to unanticipated compensating feedback. For example, adding jobs to an urban area may fail to improve unemployment if the increased attractiveness causes more people to move into the area. By emphasizing feedback and an endogenous perspective, both models help policymakers understand how policy resistance can arise. Both models challenge common beliefs about how systems work by revealing feedback loops that can exacerbate the situation, thereby facilitating learning for even the most overconfident users.”  “Finally, simulations can help to build consensus surrounding difficult policy problems. By communicating the counterintuitive nature of policy problems to policymakers, simulations can encourage dialogue and lead to the development of shared interpretations regarding the source of problem behavior. Even when different goals and value systems persist, simulation can help to focus the discussion on specific variables and outcomes that are the source of divergence.”  “We believe that small system dynamics models can contribute significantly to policymaking due to four central characteristics: first, they take a feedback approach; second, they are aggregated; third, they present simulation runs; and fourth, they are “small”. Because of these characteristics, small system dynamics models can illustrate the sources of policy resistance in the environment, facilitate learning through extensive experiments, overcome the issues of overconfidence, bring different stakeholders to a shared understanding, and help policymakers learn about the importance of an endogenous perspective to problem solving.” |
|  | 4. Group model building: Problem structuring, policy simulation and decision support – Andersen (2007) 10.1057/palgrave.jors.2602339 (*same as below Jac Vennix*) | Paper | “Furthermore as we discuss below, GMB [group model building] has much in common with six other named techniques for gaining client involvement in strategic problem finding and problem resolving activities, all using system dynamics models, none of which use the term Group Model Building.”  “System Dynamics was developed by Forrester as a way to focus on corporate policy problems (Forrester 1961)." |
| John D Sterman | 5. System dynamics: Systems thinking and modeling for a complex world – Sterman (2002) | Paper | “Today’s problems often arise as unintended consequences of yesterday’s solutions. Social systems often suffer from policy resistance, the tendency for well-intentioned interventions to be defeated by the response of the system to the intervention itself. The field of system dynamics, created at MIT [Massachusetts Institute of Technology] in the 1950s by Jay Forrester, is designed to help us learn about the structure and dynamics of the complex systems in which we are embedded, design high-leverage policies for sustained improvement, and catalyze successful implementation and change.”  “In a world of accelerating complexity and change, thoughtful leaders increasingly recognize that the tools we have been using have not only failed to solve the persistent problems we face, but may in fact be causing them. All too often, well-intentioned efforts to solve pressing problems create unanticipated side effects. Our decisions provoke reactions we did not foresee. The result is policy resistance, the tendency for interventions to be defeated by the response of the system to the intervention itself.”  “But how can one come to understand the whole system and avoid policy resistance? For many, the solution lies in systems thinking the ability to see the world as a complex system, to understand how everything is connected to everything else. With a holistic worldview, it is argued, we would be able to learn faster and more effectively, identify high leverage points, avoid policy resistance and make decisions consistent with our long-term best interests.”  “The challenge is how to move past slogans about accelerating learning and systems thinking to useful tools that help us understand complexity, design better operating policies, and guide effective change.”  “For many of the most important problems there are no purely technical solutions. Indeed, there are no purely technical problems. To be effective, engineering must consider the social, political, ecological and other impacts of proposed technical solutions.”  “Because we apply these tools to the behavior of human as well as technical systems, system dynamics also draws on cognitive and social psychology, organization theory, economics, and other social sciences. Because we strive to solve important real world problems, we must learn how to work effectively with groups of busy policy makers and how to catalyze change in organizations and in society at large.”  “This paper provides a brief overview of the system dynamics perspective and process. I briefly discuss how policy resistance arises from the mismatch between the dynamic complexity of the systems we have created and our cognitive capacity to understand that complexity. Research shows that policy resistance most often arises not because we lack the technical knowledge and tools to model the problem, but because our mental models systematically lead us to incorrect inferences about the dynamics of even the simplest systems.”  “As wonderful as the human mind is, the complexity of the world dwarfs our understanding.4 Our mental models are limited, internally inconsistent, and unreliable. Our ability to understand the unfolding impacts of our decisions is poor. We take actions that make sense from our short-term and parochial perspectives, but these decisions often feed back to hurt us in the long run. To understand the sources of policy resistance we must therefore understand both the complexity of systems and the mental models of those systems we use to make decisions.”  “Complexity is often defined in terms of the number of, or links among, the elements of a system, or the dimensionality of a search space. Such systems have high combinatorial complexity. However, most cases of policy resistance arise from dynamic complexity, the often counterintuitive behavior of complex systems that emerges from the interactions of the agents over time (Exhibit 2).”  “Counterintuitive: In complex systems cause and effect are distant in time and space while we tend to look for causes near the events we seek to explain. Our attention is drawn to the symptoms of difficulty rather than the underlying cause. High leverage policies are often not obvious.”  "Policy resistant: The complexity of the systems in which we are embedded overwhelms our ability to understand them. The result: Many seemingly obvious solutions fail or actually worsen the problem.”  “Policy resistance arises because we do not understand the full range of feedbacks operating in the system.”  “Exhibit 4 The feedback view of the world - Our decisions alter our environment, leading to new decisions, but also triggering side effects, delayed reactions, changes in goals and interventions by others. These feedbacks may lead to unanticipated results and ineffective policies.”  "System dynamics models are usually formulated as systems of high-order, nonlinear, possibly stochastic differential equations portraying the decision rules of the agents, natural processes, and physical structures relevant to the purpose of the model.”  “Zarrella asked Nick Pudar, then a senior business analyst in GM’s Corporate Strategy and Knowledge Development organization, to work on the superstore issue. Pudar told Zarrella he would need to commit to a several hour meeting each week for a month to be sure we are working on the right problem.”  “The initial modeling process dramatically redefined the problem. Superstores were only a symptom.”  “These successes show that what often prevents us from overcoming policy resistance and achieving high performance is not a lack of technical knowledge or a genuine commitment to change. Modeling takes place in the context of real world problem solving, with all its messiness, ambiguity, time pressure, politics, and interpersonal conflict. The purpose is to solve a problem, not only to gain insight (though insight is required to design effective policies).”  “As the leasing example illustrates, system dynamics, and any modeling approach, is most effective when: • it is used to solve problems, not model a system, • models have broad boundaries to capture the feedbacks, time delays, and interactions unaccounted for in people's mental models, • modelers draw on the widest array of data, both quantitative and qualitative, • other tools and methods are integrated into the effort, • the clients are actively engaged as partners in the modeling process from the start, • the model is an open box used to catalyze learning rather than a black box used to advocate policy positions, • models are open to review by all relevant stakeholders, including critics, and modelers seek out opportunities to confront the model with data and test assumptions. Such a modeling process can build both the understanding of complexity needed to find effective policies and the confidence to use that understanding to take action.” |
|  | 6. System dynamics at sixty: The path forward – Sterman (2018) 10.1002/sdr.1601 | Paper | “He did not try to build comprehensive models of a firm or economy. Instead, he built models that could be simulated and analyzed fast enough to be useful in solving specific problems.”  “There is no “correct” level of aggregation independent of model purpose. In the epidemiology example, it may seem obvious that the individual level is more correct than the aggregated level. But there is nothing uniquely privileged about the individual level: one could, in principle, further disaggregate such a model to capture the different organs and systems within each individual, or even further, to the cellular level. But why stop there? Cells and the viruses that attack them consist of still smaller structures (organelles, lipids, proteins and other molecules). Molecules consist of atoms; atoms of protons, neutrons and electrons; protons and neutrons of quarks....”  “Modelers should choose the simulation architecture and level of aggregation most appropriate for the purpose of the model—that is, best suited to solve the problem.”  “In sum, the goal of dynamic modeling is sometimes to build theoretical understanding of complex dynamic systems, sometimes to implement policies for improvement, and often both. To do so, system dynamics modelers seek to include a broad model boundary that captures important feedbacks relevant to the problem to be addressed; represent important structures in the system including accumulations and state variables, delays and nonlinearities; use behavioral decision rules for the actors and agents, grounded in first-hand study of the relevant organizations and actors; and use the widest range of empirical data to specify the model, estimate parameters, and build confidence in the results.”  “Good modelers choose the model architecture, level of aggregation and simulation method that best meet the purpose of the study, taking account of computational requirements, data availability, the time and resources available, the audience for the work, and the ability to carry out sensitivity analysis, understand the behavior of the model, and communicate the results and the reasons for them to the people they seek to influence.”  “One can go into a corporation that has serious and widely known difficulties. The symptom might be a substantial fluctuation of employment with peaks several years apart. Or the symptom might be falling market share.... In the process of finding valuable insights in the mental data store, *one talks to a variety of people in the company, maybe for many days, possibly spread over many months*. The discussion is filtered through one’s catalog of feedback structures into which the behavioral symptoms and the discussion of structure and policy might fit. The process converges toward an explicit simulation model. The policies in the model are those that people assert they are following; in fact, the emphasis is often on the policies they are following in an effort to alleviate the great difficulty. (Forrester, 1980, p. 560; emphasis added).”  “Still, Jay was confident that he could “detect the guiding policy” through observation and interviews, writing, “We usually start already equipped with enough descriptive information to begin the construction of a highly useful model” (ID, p. 57).”  “To develop reliable knowledge, we must bring the best available data to bear for the problem we seek to address. Following Jay's advice, we must find a way to measure what we need to know.”  “System dynamics models seek to capture the causal structure of a system because they are used to explore counterfactuals such as the response of a system to new policies.”  “The description violates fundamental principles for good modeling, including that one must always model a problem, never the system; that one should involve the people one hopes to influence in the modeling process from the beginning; and that modeling is iterative, with the need to revise and improve the model arising from testing (see Forrester, 1961, Appendix O and many other passages; also Sterman, 2000, ch. 3; Repenning et al., 2017).” |
|  | 7. All models are wrong: Reflections on becoming a systems scientist – Sterman (2002) 10.1002/sdr.261 | Paper | “Thoughtful leaders increasingly recognize that we are not only failing to solve the persistent problems we face, but are in fact causing them. System dynamics is designed to help avoid such policy resistance and identify high-leverage policies for sustained improvement.”  “System dynamics is also a practical tool policymakers can use to help solve important problems.”  “While it’s hard to define what system dynamics is, I don’t have any trouble answering why it is valuable. As the world changes ever faster, thoughtful leaders increasingly recognize that we are not only failing to solve the persistent problems we face, but are in fact causing them. All too often, well-intentioned efforts to solve pressing problems create unanticipated ‘‘side effects.’’ Our decisions provoke reactions we did not foresee. Today’s solutions become tomorrow’s problems. The result is policy resistance, the tendency for interventions to be defeated by the response of the system to the intervention itself. From California’s failed electricity reforms, to road building programs that create suburban sprawl and actually increase traffic congestion, to pathogens that evolve resistance to antibiotics, our best efforts to solve problems often make them worse. At the root of this phenomenon lies the narrow, event-oriented, reductionist worldview most people live by.”  “There are no side effects—only effects. Those we thought of in advance, the ones we like, we call the main, or intended, effects, and take credit for them. The ones we didn’t anticipate, the ones that came around and bit us in the rear—those are the ‘‘side effects’’. When we point to outside shocks and side effects to excuse the failure of our policies, we think we are describing a capricious and unpredictable reality. In fact, we are highlighting the limitations of our mental models. System dynamics helps us expand the boundaries of our mental models so that we become aware of and take responsibility for the feedbacks created by our decisions.”  “Failure to recognize the feedbacks in which we are embedded, the way in which we shape the situation in which we find ourselves, leads to policy resistance as we persistently react to the symptoms of difficulty, intervening at low leverage points and triggering delayed and distant, but powerful feedbacks. The problem intensifies, and we react by pulling those same policy levers with renewed vigor, at the least wasting our talents and energy, and all too often, triggering an unrecognized vicious cycle that carries us farther and farther from our goals. Policy resistance breeds a sense of futility about our ability to make a difference, a creeping cynicism about the possibility of changing our world for the better.” |
|  | 8. Learning in and about complex systems – Sterman (1994) 10.1002/sdr.4260100214 | Paper | “The challenge facing all is how to move from generalizations about accelerating learning and systems thinking to tools and processes that help us understand complexity, design better operating policies and guide organization- and society-wide learning.”  “I argue that successful approaches to learning about complex dynamic systems require (1) tools to articulate and frame issues, elicit knowledge and beliefs, and create maps of the feedback structure of an issue from that knowledge; (2) formal models and simulation methods to assess the dynamics of those maps, test new policies and practice new skills; and (3) methods to sharpen scientific reasoning skills, improve group processes and overcome defensive routines for individuals and teams, that is, in the words of Don Schön (1983a), to raise the quality of the "organizational inquiry that mediates the restructuring of organizational theory-in-use."”  “In system dynamics, 'mental model' stresses the implicit causal maps of a system we hold, our beliefs about the network of causes and effects that describe how a system operates, along with the boundary of the model (the exogenous variables) and the time horizon we consider relevant - our framing or articulation of a problem.”  “As our mental models change we create different decision rules and change the strategy and structure of our organizations. The same information, filtered and processed through a different decision rule, now yields a different decision. The development of systems thinking is a double-loop learning process in which we replace a reductionist, partial, narrow, short-term view of the world with a holistic, broad, long-term, dynamic view - and then redesign our policies and institutions accordingly.”  “Effective management is difficult in a world of high dynamic complexity. Our decisions may create unanticipated side effects and delayed consequences. Our attempts to stabilize the system may destabilize it. Our decisions may provoke reactions by other agents seeking to restore the balance we upset. Our decisions may move the system into a new regime of behavior where unexpected and unfamiliar dynamics arise because the dominant feedback loops have changed. Forrester (1971) calls such phenomena the 'counter-intuitive behavior of social systems'. It often leads to 'policy resistance', the tendency for interventions to be delayed, diluted or defeated by the response of the system to the intervention itself (Meadows 1982).”  “In practice, effective learning from models occurs best, and perhaps only, when the decision makers participate actively in the development of the model. Modeling here includes the elicitation of the participants' existing mental models, including articulating the issues (problem structuring), selecting the model boundary and time horizon, and mapping the causal structure of the relevant system.” |
|  | 9. Learning from evidence in a complex world – Sterman (2006) 10.2105/AJPH.2005.066043 | Paper | “Policies to promote public health and welfare often fail or worsen the problems they are intended to solve. Evidence-based learning should prevent such policy resistance, but learning in complex systems is often weak and slow. Complexity hinders our ability to discover the delayed and distal impacts of interventions, generating unintended “side effects.” Yet learning often fails even when strong evidence is available: common mental models lead to erroneous but self-confirming inferences, allowing harmful beliefs and behaviors to persist and undermining implementation of beneficial policies. Here I show how systems thinking and simulation modeling can help expand the boundaries of our mental models, enhance our ability to generate and learn from evidence, and catalyze effective change in public health and beyond.”  “Health care is not unique. Thoughtful leaders throughout society increasingly suspect that the policies we implement to address difficult challenges have not only failed to solve the persistent problems we face, but are in fact causing them. All too often, well-intentioned programs create unanticipated “side effects.” The result is policy resistance, the tendency for interventions to be defeated by the system’s response to the intervention itself. From overuse of antibiotics that spread resistant pathogens, to the obesity caused by the sedentary lifestyles and cheap calories our prosperity affords, our best efforts to solve problems often make them worse (box next page).”  “Policy resistance arises from a narrow, reductionist worldview. We have been trained to view our situation as the result of forces outside ourselves, forces largely unpredictable and uncontrollable. Consider the “unanticipated events” and “side effects” so often invoked to explain policy failure. Political leaders blame recession on corporate fraud or terrorism. Managers blame bankruptcy on events outside their organizations and (they want us to believe) outside their control. But there are no side effects—just effects. Those we expected or that prove beneficial we call the main effects and claim credit. Those that undercut our policies and cause harm we claim to be side effects, hoping to excuse the failure of our intervention. “Side effects” are not a feature of reality, but a sign that the boundaries of our mental models are too narrow, our time horizons too short.”  “For many, the solution is obvious: the continued application of the scientific method. The diligent adherence to scientific method, in this view, is responsible for the great advances of medicine and public health, from the Broad Street pump incident, where John Snow proved that cholera was a water-borne disease, to the latest double-blind prospective randomized clinical trial, and is the most reliable way to generate the evidence needed to improve health policy. There are, however, three fundamental impediments to this goal: the complexity problem, learning failures, and the implementation challenge. I discuss these challenges to learning from evidence in complex settings, showing how policy resistance arises from the mismatch between the complexity of the systems we have created and our capacity to understand them. I describe methods for systems thinking and formal modeling that have proven to be useful, focusing on the field of system dynamics.8,9”  “However, most cases of policy resistance arise from dynamic complexity—the often counterintuitive behavior of complex systems that arises from the interactions of the agents over time.17”  “Policy resistant. The complexity of the systems in which we are embedded overwhelms our ability to understand them. The result: many seemingly obvious solutions to problems fail or actually worsen the situation.”  “Policy resistance arises because we do not understand the full range of feedbacks surrounding—and created by—our decisions. The improvement initiatives you mandated never get off the ground because layoffs destroyed morale and increased the workload for the remaining employees. New services were rushed to market before all the kinks were worked out; unfavorable word of mouth causes the number of lucrative elective procedures to fall as patients flock to competitors. More chronically ill patients show up in your ER [emergency room] with complications after staff cuts slashed resources for patient education and follow-up; the additional workload forces still greater cuts in prevention. Stressed by long hours and continual crisis, your most experienced nurses and doctors leave for jobs with competitors, further raising the workload and undercutting quality of care. Hospital-acquired infections and preventable errors increase. Malpractice claims multiply. Yesterday’s solutions become today’s problems.”  “Ignoring the feedbacks in which we are embedded leads to policy resistance as we persistently react to the symptoms of difficulty, intervening at low leverage points and triggering delayed and distant effects.”  “Policy resistance (thick arrows) arises when we fail to account for the so called “side effects” of our actions, the responses of other agents in the system (and the unanticipated consequences of these), the ways in which experience shapes our goals, and the time delays often present in these feedbacks.”  “As our mental models change, we change the structure of our systems, creating different decision rules and new strategies. The same information, interpreted by a different model, now yields a different decision. Systems thinking is an iterative learning process in which we replace a reductionist, narrow, short-run, static view of the world with a holistic, broad, long-term, dynamic view, reinventing our policies and institutions accordingly.”  “Creating a healthy, sustainable future requires a fundamental shift in the way we generate, learn from, and act on evidence about the delayed and distal effects of our technologies, policies, and institutions. The reductionist program of ever finer specialization is no longer sufficient. Though often leading to deep and useful knowledge, it contributes to policy resistance by narrowing the boundaries of our mental models. As leaders in public health, you do not face medical problems, financial problems, technical problems, and community relations problems. You just have problems. Some boundaries are necessary and inevitable: all models must simplify the overwhelming complexity of the world. But all too often ignoring what lies outside familiar walls cuts critical feedbacks and breeds arrogance about our ability to control nature and other people—and we solve one problem only to create others.”  “What prevents us from overcoming policy resistance is not a lack of resources, technical knowledge, or a genuine commitment to change. What thwarts us is our lack of a meaningful systems thinking capability.” |
|  | 10. System dynamics modeling: Tools for learning in a complex world – Sterman (2001) 10.2307/41166098 | Paper | “More important, thoughtful leaders increasingly suspect that the tools they have been using have not only failed to solve the persistent problems they face, but may in fact be causing them. All too often, well-intentioned efforts to solve pressing problems create unanticipated side effects. Our decisions provoke unforeseen reactions. The result is policy resistance, the tendency for interventions to be defeated by the response of the system to the intervention itself.”  “However, how can one come to understand the whole system? How does policy resistance arise? How can we learn to avoid it, to find the high-leverage policies that can produce sustainable benefit? For many, the solution lies in systems thinking—the ability to see the world as a complex system, in which we understand that “you can’t do just one thing” and that “everything is connected to everything else.” With a holistic worldview, it is argued, we would be able to learn faster and more effectively, identify the high leverage points in systems, and avoid policy resistance. A systemic perspective would enable us to make decisions consistent with our long-term best interests and the long-term best interests of the system as a whole.2”  “The challenge facing us all is how to move past slogans about accelerating learning and systems thinking to useful tools that help us understand complexity, design better operating policies, and guide effective change. System dynamics is a method to enhance learning in complex systems. Just as an airline uses flight simulators to help pilots learn, system dynamics is, partly, a method for developing management flight simulators (often based on formal mathematical models and computer simulations) to help us learn about dynamic complexity, understand the sources of policy resistance, and design more effective policies.”  “To introduce this special section on system dynamics, I briefly discuss how policy resistance arises from the mismatch between the dynamic complexity of the systems we have created and our cognitive capacity to understand that complexity.”  “Policy resistance arises because, as wonderful as the human mind is, the complexity of the world dwarfs our understanding.4 Our mental models are limited, internally inconsistent, and unreliable. Our ability to understand the unfolding impacts of our decisions is poor. We take actions that make sense from our short-term and parochial perspectives, but due to our imperfect appreciation of complexity, these decisions often return to hurt us in the long run. To understand the sources of policy resistance, we must therefore understand both the complexity of systems and the mental models that we use to make decisions.”  “However, most cases of policy resistance arise from dynamic complexity— the often counterintuitive behavior of complex systems that arises from the interactions of the agents over time.”  “Characterized by Trade-Offs: Time delays in feedback channels mean the long-run response of a system to an intervention is often different from its short-run response. High leverage policies often cause worse-before-better behavior, while low leverage policies often generate transitory improvement before the problem grows worse.”  “Counterintuitive: In complex systems cause and effect are distant in time and space while we tend to look for causes near the events we seek to explain. Our attention is drawn to the symptoms of difficulty rather than the underlying cause. High leverage policies are often not obvious.”  “Policy Resistant: The complexity of the systems in which we are embedded overwhelms our ability to understand them. The result: Many seemingly obvious solutions to problems fail or actually worsen the situation.”  “Contrary to the sequential, open-loop view in Figure 1, real systems react to our interventions. There is feedback: The results of our actions define the situation we face in the future. The new situation alters our assessment of the problem and the decisions we take tomorrow (see the top of Figure 2).”  “Policy resistance arises because we do not understand the full range of feedbacks operating in the system.”  “Our decisions alter our environment, leading to new decisions, but also triggering side effects, delayed reactions, changes in goals and interventions by others. These feedbacks may lead to unanticipated results and ineffective policies.”  “To improve our ability to learn about and manage complex systems, we need tools capable of capturing the feedback processes, stocks and flows, time delays, and other sources of dynamic complexity. The tools must also enable us to understand how these structures create a system’s dynamics and generate policy resistance. They must help us evaluate the consequences of new policies and new structures we might design. These tools include causal mapping and simulation modeling.”  “They show that many policies undertaken to escape the trap— including many programs to implement new product development processes and tools—are self-defeating, and they explore effective policies to overcome the trap.”  “These successes show that what often prevents us from overcoming policy resistance and achieving high performance is not a lack of resources, technical knowledge, or a genuine commitment to change. What thwarts us is our lack of a meaningful systems-thinking capability, the capability to learn about complexity and find the high leverage policies through which we can create the future we truly desire.” |
|  | 11. Business Dynamics – Sterman (2000) | Book | “Many of the problems we now face arise as unanticipated side effects of our own past actions. All too often the policies we implement to solve important problems fail, make the problem worse, or create new problems. Effective decision making and learning in a world of growing dynamic complexity requires us to become systems thinkers-to expand the boundaries of our mental models and develop tools to understand how the structure of complex systems creates their behavior.”  “The goal of systems thinking and system dynamics modeling is to improve our understanding of the ways in which an organization’s performance is related to its internal structure and operating policies, including those of customers, competitors, and suppliers and then to use that understanding to design high leverage policies for success.”  “System dynamics is also designed to be a practical tool that policy makers can use to help them solve the pressing problems they confront in their organizations.”  “All too often, well-intentioned efforts to solve pressing problems lead to policy resistance, where our policies are delayed, diluted, or defeated by the unforeseen reactions of other people or of nature. Many times our best efforts to solve a problem actually make it worse.”  “System dynamics is a method to enhance learning in complex systems. Just as an airline uses flight simulators to help pilots learn, system dynamics is, partly, a method for developing management flight simulators, often computer simulation models, to help us learn about dynamic complexity, understand the sources of policy resistance, and design more effective policies.”  “Because we build system dynamics models to solve important real world problems, we must learn how to work effectively with groups of busy policy makers and how to catalyze sustained change in organizations.”  “From Thomas More in 1516 to Pogo in the mid 20th century it has long been acknowledged that people seeking to solve a problem often make it worse. Our policies may create unanticipated side effects. Our attempts to stabilize the system may destabilize it. Our decisions may provoke reactions by others seeking to restore the balance we upset. Forrester (1971a) calls such phenomena the “counterintuitive behavior of social systems.” These unexpected dynamics often lead to policy resistance, the tendency for interventions to be delayed, diluted, or defeated by the response of the system to the intervention itself (Meadows 1982).” (**1.1.1 Policy Resistance, the Law of Unintended Consequences, and the Counterintuitive Behavior of Social Systems**)  “Policy resistance arises because we often do not understand the full range of feedbacks operating in the system (Figure 1.4). As our actions alter the state of the system, other people react to restore the balance we have upset. Our actions may also trigger side effects.”  “To avoid policy resistance and find high leverage policies requires us to expand the boundaries of our mental models so that we become aware of and understand the implications of the feedbacks created by the decisions we make. That is, we must learn about the structure and dynamics of the increasingly complex systems in which we are embedded.”  “In system dynamics, the term “mental model” includes our beliefs about the networks of causes and effects that describe how a system operates, along with the boundary of the model (which variables are included and which are excluded) and the time horizon we consider relevant-our framing or articulation of a problem.”  “The type of reframing stimulated by Fred’s intervention, denoted double-loop learning by Argyris (1985), is illustrated in Figure 1.11. Here information feedback about the real world not only alters our decisions within the context of existing frames and decision rules but also feeds back to alter our mental models. As our mental models change we change the structure of our systems, creating different decision rules and new strategies. The same information, processed and interpreted by a different decision rule, now yields a different decision. Altering the structure of our systems then alters their patterns of behavior. The development of systems thinking is a double-loop learning process in which we replace a reductionist, narrow, short-run, static view of the world with a holistic, broad, long-term, dynamic view and then redesign our policies and institutions accordingly.”  “Counterintuitive: In complex systems cause and effect are distant in time and space while we tend to look for causes near the events we seek to explain. Our attention is drawn to the symptoms of difficulty rather than the underlying cause. High leverage policies are often not obvious. whelms our ability to understand them. The result: Many seemingly obvious solutions to problems fail or actually worsen the situation. Characterized by trade-offs: Time delays in feedback channels mean the long-run response of a system to an intervention is often different from its short-run response. High leverage policies often cause worse-before-better behavior, while low leverage policies often generate transitory improvement before the problem grows worse.”  “Many of the tools of system dynamics are designed to help you develop useful, reliable, and effective models to serve as virtual worlds to aid learning and policy design.”  “A central principle of system dynamics is to examine issues from multiple perspectives; to expand the boundaries of our mental models to consider the long-term consequences and “side effects” of our actions, including their environmental, cultural, and moral implications (Meadows, Richardson, and Bruckmann 1982).”  “Complex dynamic systems present multiple barriers to learning. The challenge of bettering the way we learn about these systems is itself a classic systems problem. System dynamics is a powerful method to gain useful insight into situations of dynamic complexity and policy resistance. It is increasingly used to design more successful policies in companies and public policy settings.”  “Pudar told Zarella he would need to commit to a several hour meeting each week for a month “to be sure we are working on the right problem.””  “Opening the model to review by the clients is also essential for the modelers to ensure it addresses the issues the client cares most deeply about and to generate the best model for that purpose.”  “Though the projects described above differed in many ways, they all illustrate a number of principles for effective development and implementation of system dynamics models (see chapter 3; see also Forrester 1961; Roberts 1977/1978; and Morecroft and Sterman 1994): 1. Develop a model to solve a particular problem, not to model the system. A model must have a clear purpose and that purpose must be to solve the problem of concern to the client. Modelers must exclude all factors not relevant to the problem to ensure the project scope is feasible and the results timely. The goal is to improve the performance of the system as defined by the client. Focus on results.”  “5. Focus on implementation from the start of the project. Implementation must start on the first day of the project. Constantly ask, How will the model help the client make decisions? Use the model to set priorities and determine the sequence of policy implementation. Use the model to answer the question, How do we get there from here? Carefully consider the real world issues involved in pulling various policy levers. Quantify the full range of costs and benefits of policies, not only those already reported by existing accounting systems. 6. Modeling works best as an iterative process of joint inquiry between client and consultant. Modeling is a process of discovery. The goal is to reach new understanding of how the problem arises and then use that understanding to design high leverage policies for improvement. Modeling should not be used as a tool for advocacy. Don’t build a client’s prior opinion about what should be done into a model. Use workshops where the clients can test the model themselves, in real time.” (**2.5 SUMMARY: PRINCIPLES FOR SUCCESSFUL USE OF SYSTEM DYNAMICS**)  “Modeling takes place in the context of real world problem solving, with all its messiness, ambiguity, time pressure, politics, and interpersonal conflict. The purpose is to solve a problem, not only to gain insight (though insight into the problem is required to design effective policies).”  “There is clearly a role for models that help managers pilot their organizations better, and system dynamics is often useful for these purposes. But the real value of the process comes when models are used to support organizational redesign. In Industrial Dynamics, Forrester calls for courage in the selection of problems, saying, “The solutions to small problems yield small rewards. . . The goal should be to find management policies and organizational structures that lead to greater success.” Focus your modeling work on the important issues, on the problems where your work can have lasting benefit, on the problems you care most deeply about.”  “The client context and real world problem determine the nature of the model, and the modeling process must be consistent with the clients’ skills, capabilities, and goals. The purpose is to help the clients solve their problem.”  “Table 3.1. Steps of the modeling process. 1. Problem Articulation (Boundary Selection) – Theme selection: What is the problem? Why is it a problem?”  “5. Policy Design and Evaluation – (…) Policy design: What new decision rules, strategies, and structures might be tried in the real world? How can they be represented in the model?”  “Yet all successful modelers follow a disciplined process that involves the following activities: (1) articulating the problem to be addressed, (2) formulating a dynamic hypothesis or theory about the causes of the problem, (3) formulating a simulation model to test the dynamic hypothesis, (4) testing the model until you are satisfied it is suitable for your purpose, and (5) designing and evaluating policies for improvement.”  “The initial purpose dictates the boundary and scope of the modeling effort, but what is learned from the process of modeling may feed back to alter our basic understanding of the problem and the purpose of our effort.”  “The most important step in modeling is problem articulation. What is the issue the clients are most concerned with? What problem are they trying to address? What is the real problem, not just the symptom of difficulty? What is the purpose of the model? A clear purpose is the single most important ingredient for a successful modeling study. Of course, a model with a clear purpose can still be misleading, unwieldy, and difficult to understand. But a clear purpose allows your clients to ask questions that reveal whether a model is useful in addressing the problem they care about. Beware the analyst who proposes to model an entire business or social system rather than a problem. Every model is a representation of a system-a group of functionally interrelated elements forming a complex whole. But for a model to be useful, it must address a specific problem and must simplify rather than attempt to mirror an entire system in detail. What is the difference? A model designed to understand how the business cycle can be stabilized is a model of a problem. It deals with a specific policy issue. A model designed to explore policies to slow fossil fuel use and mitigate global warming is also a model of a problem; it too addresses only a limited set of issues. A model that claims to be a representation of the entire economy is a model of a whole system. Why does it matter? The usefulness of models lies in the fact that they simplify reality, creating a representation of it we can comprehend. A truly comprehensive model would be just as complex as the system itself and just as inscrutable. Von Clausewitz famously cautioned that the map is not the territory. It’s a good thing it isn’t: A map as detailed as the territory would be of no use (as well as being hard to fold). The art of model building is knowing what to cut out, and the purpose of the model acts as the logical knife. It provides the criteria to decide what can be ignored so that only the essential features necessary to fulfill the purpose are left. In the example above, since the purpose of the comprehensive model would be to represent the entire economic system, nothing could be excluded. To answer all conceivable questions about the economy, the model would have to include an overwhelming array of variables. Because its scope and boundary are so broad, the model could never be completed. If it were, the data required to use it could never be compiled. If they were, the model’s underlying assumptions could never be examined or tested. If they were, the model builders could never understand its behavior and the clients’ confidence in it would depend on the authority of the modeler and other nonscientific grounds. Mihailo Mesarovic, a developer of early global simulations, captured the impossibility of building models of systems when he said, “No matter how many resources one has, one can envision a complex enough model to render resources insufficient to the task.” (Meadows, Richardson, and Bruckmann 1982, p. 197). A model designed for a particular purpose such as understanding the business cycle or global climate change would be much smaller, since it would be limited to those factors believed to be relevant to the question at hand. For example, the business cycle model need not include long-term trends in population growth, resource depletion, or climate change. The global warming model could exclude short-term dynamics related to interest rates, employment, and inventories. The resulting models could be simple enough so that their assumptions could be examined. The relation of these assumptions to the most important theories regarding the business cycle and climate change could then be assessed to determine how useful the models were for their intended purposes. Of course even models with well-defined purposes can be too large. But without a clear purpose, there is no basis to say “we don’t need to include that” when a member of the client team makes a suggestion. In sum: Always model a problem. Never model a system.” (**3.5.1 Problem Articulation: The Importance of Purpose**)  “To do so you and the clients must identify the time horizon and define those variables and concepts you consider to be important for understanding the problem and designing policies to solve it.”  “A principal deficiency in our mental models is our tendency to think of cause and effect as local and immediate. But in dynamically complex systems, cause and effect are distant in time and space. Most of the unintended effects of decisions leading to policy resistance involve feedbacks with long delays, far removed from the point of decision or the problem symptom. Work with your clients to think about the possible reactions to policies and how long they might take to play out and then increase the time horizon even further.”  “System dynamics seeks endogenous explanations for phenomena. The word “endogenous” means “arising from within.” An endogenous theory generates the dynamics of a system through the interaction of the variables and agents represented in the model. By specifying how the system is structured and the rules of interaction (the decision rules in the system), you can explore the patterns of behavior created by those rules and that structure and explore how the behavior might change if you alter the structure and rules.”  “Once you and the client have developed confidence in the structure and behavior of the model, you can use it to design and evaluate policies for improvement. Policy design is much more than changing the values of parameters such as a tax rate or markup ratio. Policy design includes the creation of entirely new strategies, structures, and decision rules. Since the feedback structure of a system determines its dynamics, most of the time high leverage policies will involve changing the dominant feedback loops by redesigning the stock and flow structure, eliminating time delays, changing the flow and quality of information available at key decision points, or fundamentally reinventing the decision processes of the actors in the system.” (**3.5.5 Policy Design and Evaluation**)    “Models must be clearly focused on a purpose. Never build a model of a system. Models are simplifications; without a clear purpose, you have no basis for excluding anything from your model and your effort is doomed to failure. Therefore the most important step in the modeling process is working with your client to articulate the problem-the real problem, not the symptoms of the problem, the latest crisis, or the most recent fad. Of course, as the modeling process leads you to deeper insight, your definition and statement of the problem may change. Indeed, such radical reframings are often the most important outcome of modeling. The purpose of modeling is to help the clients solve their problem. Though the modeling process often challenges the clients’ conception of the problem, ultimately, if the client perceives that your model does not address their concern, you can have little impact. The modeler must not grow attached to a model, no matter how elegant or how much time has been invested in it. If it doesn’t help the clients solve their problem, it needs to be revised until it does.”  “By showing the network of feedback loops in which policies are embedded, causal diagrams are often an effective way to show how event-oriented, open-loop mental models lead to policy resistance.”  “The real value of modeling is not to anticipate and react to problems in the environment but to eliminate the problems by changing the underlying structure of the system.”  “The problem was not generating ideas but evaluating which ideas might work, how they might interact, which would have the highest leverage, and which should be implemented first.”  “Once we recognize that all models are wrong and abandon the black and white dualism of truth and falsification, we can focus on the important questions: Is the model useful? Do its shortcomings matter? To answer these questions you must first ask: Useful for what purpose? Matter to whom? Model users must critically assess the model’s boundary, time horizon, and level of aggregation in light of their purpose. The model boundary determines which variables are treated endogenously, which are treated exogenously, and which are excluded altogether. Factors relevant to the purpose must be captured endogenously. Treating a concept as exogenous, or omitting it, cuts all feedbacks involving that variable. Models with narrow boundaries don’t capture the system’s responses to policies, leaving the clients to discover them as unforeseen side effects in the real world. Narrow model boundaries are the single greatest source of policy resistance in systems (Chapter 1).”  “Table 21.1. Questions model users should ask – but usually don’t. Purpose, Suitability, and Boundary What is the purpose of the model? What is the boundary of the model? Are the issues important to the purpose treated endogenously? What important variables and issues are exogenous, or excluded? Are important variables excluded because there are no numerical data to quantify them? What is the time horizon relevant to the problem? Does the model include the factors that may change significantly over the time horizon as endogenous elements? Is the level of aggregation consistent with the purpose?”  “The ultimate goal of modeling is to solve a problem. System improvement tests ask whether the modeling process helped change the system for the better. To pass the test, the modeling process must identify policies that lead to improvement, those policies must be implemented, and the performance of the system must actually improve.” |
|  | 12. Systems thinking and organizational learning: Acting locally and thinking globally in the organization of the future – Senge (1992) 10.1016/0377-2217(92)90011-w | Paper | “The challenge is how to move from generalizations about accelerating learning and systems thinking to tools and processes that help managers reconceptualize complex issues, design better operating policies and guide organization-wide learning.”  “Mapping tools serve many purposes. They assist in generating issues, capturing and framing knowledge, sharing concepts, focussing discussion, and reaching consensus.”  “The key to the process is the discipline imposed by the modelling tools. Ideas for improvement must be translated into specific changes in policy and structure.”  “Moreover, the model implied that responsibility for the insurance crisis rests in part with established management practices, when most within the firm regard the problem as externally caused. Specifically, the model suggested that established policies had produced declining quality and increasing settlement size - precisely the opposite of the organization's lofty vision and espoused policies. The problem now facing the team was how to develop shared understanding throughout the organization. The managers who went through the intense learning process could not expect those who had not to agree with its 'counterintuitive' implications.” |
| Khalid Saeed | 13. Slicing a complex problem for system dynamics modelling – Saeed (1992) 10.1002/sdr.4260080305 | Paper | “There are many ways to slice a complex problem but not all create partitions that keep together processes contributing to effective policy design. This paper explores ways in which a complex problem may be appropriately sliced so the models of the partitions can serve as effective tools for policy design.”  “Models cannot be made overly complex if they are to remain understandable. Therefore, complex problems must be sliced into smaller parts in a way that the parts meet the requirements of intended policy design. There exist many ways to partition a problem, although not all models so created are useful for identifying sensitive entry points for system change that a system dynamics modelling effort must seek. This paper attempts to outline a problem slicing process that should facilitate creating relatively simple models without disconnecting symbiotic processes in a system contributing to change, so policy design can be attempted in parts without loss of value.”  “Such a model, although precisely tracking a particular history, may be so closely tied to the specific behavioral pattern it reproduces that it may not contain the policy space for designing any mechanisms of change. Unfortunately, there is little utility for such modelling except forecasting, the policy implications of which are direct exogenous intervention. In my observation, many system dynamics models are being built in this way, creating interventionist policy agenda for fighting internal trends rather than changing them, which is considerably removed from the classical system dynamics perspective.”  “Thus, intuitively sensible schemes of partitioning a system may often create sub-models that do not incorporate policy space for attempting a design of change.”  “As they are enlarged, such models will be able to track history more and more precisely, although they will rarely incorporate any organizational means for changing the patterns they replicate.”  “A model that is suitable for designing a change must correspond to an equifinal system which can assume many patterns of behavior [Bertalanffy 1968, Katz & Kahn 1978].”  “b) The dynamic hypothesis should address the issue of change not merely the creation of local patterns. Thinking about change must prevail upon all modelling details starting with the dynamic hypothesis designating the model boundary and identifying the key feedback loops underlying the behavioral patterns described by the reference mode.”  “The guidelines for the policy design, therefore, are to be conceived in terms of either the new feedback loops that must be created to modify the anatomy of a critical decision or the way the influence structure of the existing feedback loops is to be changed so that the dominance of insidious mechanisms is minimized and the role of benign mechanisms is enhanced.” |
|  | Development Planning and Policy Design: A System Dynamics Approach – Saeed (1994) | Book | *Unavailable.* |
| Navid Ghaffarzadegan | How small system dynamics models can help the public policy process – Ghaffarzadegan (2010) 10.1002/sdr.442 | Paper | *Same as above.* |
| Jack B Homer | 14. System dynamics modeling for public health: Background and opportunities – Homer (2006) 10.2105/AJPH.2005.062059 | Paper | “The system dynamics approach involves the development of computer simulation models that portray processes of accumulation and feedback and that may be tested systematically to find effective policies for overcoming policy resistance.”  “Many of these complex problems have persisted for decades, often proving resistant to attempts to solve them.4”  “Dynamically complex problems are often characterized by long delays between causes and effects, and by multiple goals and interests that may in some ways conflict with one another. In such situations, it is difficult to know how, where, and when to intervene, because most interventions will have unintended consequences and will tend to be resisted or undermined by opposing interests or as a result of limited resources or capacities.”  “The methodology involves development of causal diagrams and policy-oriented computer simulation models that are unique to each problem setting.”  “System dynamics uniquely offers the practical application of these concepts in the form of computerized models in which alternative policies and scenarios can be tested in a systematic way that answers both “what if” and “why.”14–16”  “These numerous requirements help to ensure that a model is reliable and useful not only for studying the past, but also for exploring possible futures.12,17”  “System dynamics modelers find that a broad boundary including a variety of realistic causal factors, policy levers, and feedback loops is often what is needed for finding effective solutions to persistent, dynamically complex problems.7,53”  “The value of system dynamics modeling is best explained by way of illustration. We start with a challenging question: Why is it that, despite repeated calls for a greater emphasis on primary prevention of chronic disease (including a prominent recent example54), the vast majority of health activities and expenditures in the United States are made not for such prevention but rather for disease management and care?55 This dominance of “downstream” over “upstream” health activities appears to have grown ever greater during the era of modern medicine and is now seen as a pressing problem by public health agencies such as the Centers for Disease Control and Prevention (CDC).56 To illustrate how system dynamics simulation might shed light on this question, we have built a relatively simple model exploring how a hypothetical chronic disease population may be affected by 2 types of prevention: upstream prevention of disease onset, and downstream prevention of disease complications.”  “Figure 1 presents the model’s essential causal structure and policy inputs.”  “System dynamics models could also address multiple interacting diseases and risks, giving a more realistic picture of their overall epidemiology and policy implications, particularly where the diseases and risks are mutually reinforcing. For example, it has been found that substance abuse, violence, and AIDS often cluster in the same urban subpopulations, and that such “syndemics” are resistant to narrow policy interventions.62–64” |
| Jac Vennix | 15. System dynamics and organizational interventions – Rouwette (2006) 10.1002/sres.772 | Paper | “The main reason for involving clients is that, although system dynamics models can be used productively to analyse strategic problems and to come up with robust strategies this does not mean that the proposed strategies will automatically be adopted by the organization. This may be due to the fact that the model does not deal adequately with prevailing discussions on policies in a company.”  “According to Andersen et al. (1997, p. 190), a research programme on group model building will need to be based on a clear idea of the intended outcomes of the intervention.”  “The authors describe a number of goals at the individual level: learning or mental model improvement, change in attitudes and change in behaviour. At the group level they list three goals: mental model alignment, consensus and commitment to a decision. The ultimate outcomes of modelling are formulated at the level of the organization. Constructing a model of an organizational problem is expected to lead to system changes (doing things differently) and system results (improvement of the problematic condition).”  “It is worth noting that placing goals in a single list might confer the erroneous message that goals are equally important across all modelling projects. This is probably a simplification. Depending on the situation or modeller, specific goals might be emphasized and others ignored.”  “Depending on the side favoured in these discussions a system dynamicist will tend to choose a particular approach to modelling a client’s problem. Zagonel (2002, 2004) argues that these debates are based on a more fundamental issue, which is the desire to create models that represent reality (the micro world view) or align views of problem stakeholders (the boundary object view).”  “The core practices in modelling which all participants identified as important, can be summarized as follows: - Problem identification and definition: talk and listen reflectively to clients, clarify the purpose, identify the reference mode, ask why current behaviour is generated, formulate the dynamic hypothesis.”  “If the purpose of the model is (partly) to operate as a boundary object, the process of modelling needs to accommodate negotiation between stakeholders and integration of partial views.”  “If we combine the considerations on the design of a group model building project offered by several authors (Vennix et al., 1994; Vennix, 1996; Andersen and Richardson, 1997; Luna- Reyes and Andersen, 2003; Richardson et al., 2004) the following seem to be important: - suitability of system dynamics for the client’s problem; - purpose of the modelling effort; - clarity of the client’s problem; (…)”  “According to Zagonel the model is more likely to be used as a boundary object if there are widely divergent views on the problem. Campbell (2001) and Winch and Derrick (this issue) describe projects that started from a broad problem definition. Both projects passed through a stage of ‘confusion’ before arriving at a more structured view of the problem. In a situation where the problem is vague and a group is just beginning to address a problem, Vennix et al. (1994) recommend to use an open format for modelling sessions.”  “If the context and requirements of a project (suitability of system dynamics, project purpose, clarity and participants) are clear, there is still a lot of leeway in setting up a particular group session.”  “Scripts for defining a problem for instance include presenting reference modes, eliciting reference modes and clarifying audience, purpose and policy options.”  “In total nine domains of outcomes are measured: the modelling team’s assessment of the intervention, participants’ reaction to the sessions, shifts in goals and strategies, alignment of mental models, shifts in understanding how the system functions, and finally three types of shifts in system structure. With the exception of shifts in strategies and two types of structural change, again outcomes are positive.”  “Richardson et al. (1994) separate mental models into means, ends and means-ends models. The ends model contains goals, while the means model consists of strategies, tactics and policy levers. The means-ends model contains the connection between the two former types of models and may contain either detailed ‘design’ logic or more simple ‘operator’ logic. The three types of models are derived from the psychological research by Brunswik (1955, Hammond and Stewart, 2001). On the basis of research on participants in management flight simulators (Richardson and Rohrbaugh, 1990; Andersen et al. 1994), the authors conclude that operator logic, or high-level heuristics, is a necessary condition for improving system performance. Therefore providing managers with operator knowledge is the key to implementation of system changes. In the field experiment mentioned earlier, Huz and colleagues (Huz et al., 1997; Huz, 1999) find significant shifts in participants’ goal structures, change strategies and understanding of how the system functioned.”  “The identification of levers for steering problematic behaviour in the right direction and simulation of proposed interventions under a range of exogenous scenarios, are likely to identify and circumvent barriers to actions.”  “Many system dynamics practitioners explicitly connect their intervention to decision making biases and shortcomings (e.g. Sterman, 1994; Vennix, 1996).” |
|  | 16. Group model-building: Tackling messy problems – Vennix (1999) 10.1002/(SICI)1099-1727(199924)15:4%3C379::AID-SDR179%3E3.0.CO;2-E | Paper | “Almost since its inception, system dynamicists have involved the client (groups) in the model-building process for at least three reasons. First, to capture the required knowledge in the mental models of the client group (Forrester 1961; 1987). Second, to increase the chances of implementation of model results (cf. Roberts 1978; Weil 1980), and, finally, to enhance the client's learning process (Greenberger et al. 1976; de Geus 1988; Lane 1989; Morecroft 1992; Morecroft and Sterman 1992).”  “Others have employed Group Model-Building interventions to work with management teams on less tangible, ill-defined strategic issues, labelled by some scholars as messy problems (Ackoff 1974; 1979), i.e., situations in which there are large differences of opinion on the problem or even on the question of whether there is a problem.2 In these cases the emphasis is necessarily, but not exclusively, on problem structuring and on creating consensus and commitment with a group decision (Lane 1992; 1993; Wolstenholme 1990, 1992, 1999; Vennix 1996; Majone 1984; Zakay 1984), in order for concerted action to result (Drucker 1988).”  “In addition, experiments in dynamic decision making have revealed that people tend to ignore feedback processes, which produces detrimental results (Sterman 1989a; 1989b; 1994; Brehmer 1989; Kleinmuntz 1993), a result that is confirmed by field research (Hall 1984). And again explicit training in understanding the feedback structure of the system has virtually no impact on people's ability to manage such a system effectively (Maxwell et al. 1994; Richardson et al. 1994).”  “Many Group Model Building and systems thinking interventions work on this assumption and consider simulation to be the primary contribution to the improvement of a group's information processing capacity.”  “Group model building and system dynamics help to uncover these illusions, because the rigour of mapping and modelling forces participants to carefully and consistently make their mental models explicit and put their problem definitions to the test, by surfacing implicit (causal) assumptions.”  “Second, people's mental models are frequently only partial representations of a complex situation. Managers tend to see parts rather than wholes, particularly when they are not trained in systems thinking (cf. Dearborn and Simon 1957; Hall 1984). The result is departmental bias and potential deadlock situations, which may jeopardise organised action and may even lead to the demise of the organisation (Hall, 1984). Group Model-Building not only offers an opportunity to share and align piecemeal mental models (Huz et al. 1997), but also creates the possibility of assimilating and integrating partial mental models into a holistic system description, making participants overcome their local, departmental views (Vennix 1995; 1996).”  “System dynamicists have repeatedly pointed out the flaws in human mental models and blame many of the problems in policy making to these deficiencies. Much of the literature in system dynamics (tacitly) assumes that better understanding of the structure and behaviour of complex systems by employing system dynamics simulation will solve most of these problems.” |
|  | Group model building: Problem structuring, policy simulation and decision support – Andersen (2007) 10.1057/palgrave.jors.2602339 | Paper | *Same as above.* |
|  | Group Model Building – Vennix (1996) | Book | *Unavailable.* |
|  | 17. On evaluating the performance of problem structuring methods: An attempt at formulating a conceptual model – Rouwette (2007) 10.1007/s10726-007-9100-z | Paper | “In contrast, the model-based approach originates in Operational Research/Management Science and focuses on the different sorts of decision models (Morton et al. 2003). Researchers working in the model-based tradition are concerned with the understanding of a phenomenon in its context and from the perspective of participants. Research is interpretivistic in nature and often takes the form of action research. Finlay (1998, p. 195) points to subjective idealism and normativism as the underlying philosophy of PSMs [problem structuring methods]. Besides understanding the perspective of the participants, PSM proponents aim to help a group in deciding how to act and support the creation of commitment to future actions (Eden 1995). In contrast to offering a product, ‘… model-driven researchers are more likely to see themselves as offering a ‘problem structuring’ (…) or ‘problem consultation’ (…) *service*, a vital component of which is the skill of the change agent’ (Morton et al. 2003, p. 115, italics in original).”  “He feels there is no common set of goals for all PSMs [problem structuring methods], but a specific PSM should be clear about its assumptions with regard to these three subjects. In their overview of PSM research, Morton et al. (2003) see the following important explanatory variables: the type of model, various dimensions of facilitation, the role of the client and the stage of decision making (for example divergent–convergent). Important process variables are negotiation (of agenda and meaning), elicitation and accommodation of problem views. Central outcomes are commitment to future action and learning.”  “Eden (1989, p. 25) describes the central role of concepts in structuring problems and ‘language as the currency of organizational life’. Friend (2001, p. 120) sees the support of communication among decision makers as the main purpose of problem structuring. Eden (1992, p. 205) identifies information exchange and egalitarianism as two central elements of PSMs [problem structuring methods]. He also seems to refer to different paths for changing mental models in the following (Eden 1992, p. 208): “In group decision making we expect to see a shift in emotional attitudes as well as a cognitive shift to the problem situation. Changes in emotional attitude reflect, in part, the role of intuition and hunch which leads to a feeling of comfort about the path ahead (…). Cognitive shifts are about someone “changing their mind” – changed beliefs, changed values, and changes in the salience of particular values (…) As I have argued above, it is more likely that the procedural rationality will influence emotional attitudes, and substantial rationality will influence shifts in cognition; however, each supports the other.””  “Procedural rationality is concerned with following the proper process, while substantive rationality refers to the arguments that can be brought to bear on a person’s position. Checkland (1989, p. 83) refers to negotiation and debate as the vehicles through which people learn and arrive at shared perceptions. Both Checkland and Eden refer to the comparison of individual models to achieve learning. It seems that SSM [soft systems methodology] and SODA/Journey making attend to facilitated (egalitarian) discussion in order to achieve changes in participants’ ideas and goals (mental models) (see also Pidd 2003). Checkland (2000, p. S33) for example points to two foci of SSM that inform each other: the sensemaking and action-oriented orientation. Changes in mental models are expected to contribute to consensus and commitment to future actions.”  “Most importantly, PSMs [problem structuring methods] and group model building share a focus on people’s perceptions (Woolley and Pidd 1981). Rosenhead (1989) and Rosenhead and Mingers (2001, p. 11) describe a number of characteristics of methods for problem structuring: the approaches seek alternative solutions which are acceptable on separate dimensions (not focusing on optimization), integrate hard and soft data with social judgments, are simple and transparent, view people as active subjects, facilitate bottom-up planning and accept uncertainty (see also Ackermann and Eden with Brown 2005; Eden and Ackermann 2006).”  “Zagonel (2004) concludes that models constructed in group model building sessions have a dual identity: at some points in the modeling process they are seen as descriptions of the real world (micro-worlds) and at other times as socially constructed artifacts (boundary objects).”  “The two generic goals of PSM [problem structuring], commitment to future action and learning (Morton et al. 2003), can also be found in the literature on group model building. Forrester’s founding ideas of system dynamics (1958, 1961) include an integrative perspective on material and information flows and decisions, and the explanation of system behavior from the closed loops between the state of the system and stakeholders’ decisions. The ultimate purpose of system dynamics interventions is then to improve the performance of the system, and it was readily recognized that stakeholders’ opinions, convictions or ideas on system functioning are crucial in accomplishing this. Stakeholders’ mental models include important information on the issue of concern, part of which cannot be found in other information sources (Forrester 1961; Morecroft 2004). Mental models do not only provide information on the functioning of the system as a whole, but are also the base for an individual stakeholder’s actions. Policies within the larger system are founded on the decision maker’s limited store of information and decision rules. The mental model determines which system variables are scanned for information as well as the goal to be reached. It is therefore not surprising that mental models are central to modeling practice. Doyle and Ford (1998, p. 4) formulate this as follows: “Mental models are thus the stock in trade of research and practice in system dynamics: they are the “product” that modelers take from students and clients, disassemble, reconfigure, add to, subtract from, and return with value added.””  “Richardson et al. (1994) distinguish three elements of a mental model. A representation of prerequisites for actions such as strategies, tactics and policy levers is referred to as a means model. The ideas on the dynamic system are stored in a means/ends model. The ends model contains goals.”  “In many descriptions of the phases of modeling (e.g. Richardson and Pugh 1981), the implementation of system changes forms the final step. However, more and more authors describe implementation as a goal pervading the complete process of model construction (e.g. Roberts 1978; Vennix et al. 1996). It seems logical to expect system improvement to be a goal even higher in the hierarchy, for which commitment and implementation are a prerequisite.”  “It is worth noting that placing goals in a single list might confer the erroneous message that goals are equally important across all modeling projects. This is probably a simplification. Depending on the situation or modeler, specific goals might be emphasized and others ignored (Zagonel 2004).”  “The mainstream of publications on system dynamics methodology is however not concerned with group decision making or facilitation, but instead focuses on model content: analysis of structure and behavioral patterns, validation and testing, and policy experimentation. The system dynamics model and participants’ mental models are expected to be closely related. Most system dynamicists would probably consider the development and analysis of a model as the main vehicle to produce insight. This resembles the view of models as transitional objects or items people can play with in order to refine their understanding of a particular subject (Morecroft 1992; Zagonel 2004; see also Eden and Ackermann 2001b). Lane (1992, p. 74) sees the function of models as making the view of participants more coherent: ‘…goals which seemed reasonable when only part of the system was viewed are seen as inconsistent or impossible in the context of the whole system.’”  “In a more general sense, Vennix et al. (1996); Vennix (1999) relates the construction of a system dynamics model to individual perception and retention processes. The human information processing capacity cannot deal adequately with complex systems, as humans are biased in their decision making and fail to see feedback processes (Sterman 1994). A model helps participants to structure the problem and enables them to put their problem definitions to the test.” |
| Robert Y Cavana | 18. Integrating critical thinking and systems thinking: From premises to causal loops – Cavana (2004) 10.1002/sdr.294 | Paper | “This article demonstrates how to construct a policy argument from the perspective of critical thinking and how to convert this into a causal loop diagram utilising the tools of systems thinking and system dynamics. It is based on the authors’ recent work on the Demonstrating Values project with the New Zealand Customs Service, which involved integrating critical thinking and systems thinking into the policy development process. Critical thinking is used in this context to reconstruct a rationale for a given policy. This helped us to isolate key concepts and desired outcomes. The concepts were then used to construct a conceptual diagram. Based on this, the key concepts were operationalised to form a causal loop diagram including the main variables of interest in the system. The causal loop diagram can then be used as the basis for further policy interventions and analysis.”  “In the assessment of the activities of an organisation, managers have to link an outcome with the organisation’s goals. In certain cases the reasoning that connects an action (or policy) with its supposed purpose can be complicated. In these cases the link can seem quite clear but when the reasoning is examined closely, the links can appear much more tenuous than originally thought. What we suggest doing, and have done with the New Zealand Customs Service, is to reconstruct the reasoning behind the implementation of an action (or policy) to see whether the reasoning (1) really does warrant taking that action and (2) whether there are other alternative courses of action that will better serve the purpose of the action. Critical thinking is used in this context as a means for constructing a justification of an action (or policy) in terms of a deductive argument.”  “The action in question is that the NZCS [New Zealand Customs Service] take effective measures against the entry of anthrax into New Zealand. The conclusion of our argument, since it is supposed to be a justification of this action, is that this action should take place. When constructing an argument of this form, we use common sense and the expertise of people in the organisations to determine the reasoning behind the proposed action. One might wonder why we do not merely ask the officials in charge of the policy why they have put it in place. Sometimes this is possible but often it is inconvenient and, more often, the people responsible for the policy do not have explicit reasons for policies—they too would have to reconstruct them in the same way. Here is the reconstructed argument. The argument is done in terms of several sub-arguments. It is important that every step in the argument be made explicit. This is the point of the exercise—to bring out every assumption behind a policy decision.”  “The learning outcome from this exercise was to provide a better understanding of the dynamic issues, short- and long-term consequences of actions and policies, a better understanding of system behaviour, and possible intervention points in the system to improve behaviour.”  “We suggest using critical thinking to help develop rationale for policy. Then we explained how to convert premises and arguments to form a conceptual diagram and subsequently a causal loop diagram, which can be used as the basis for developing actions and implementation of policy. From the standpoint of causal loop diagrams, critical thinking provides a way into how concepts can be operationalised. From the standpoint of critical thinking, systems thinking and causal loop diagramming provide a way of moving from a conceptual understanding of a policy to implementation (i.e., from thinking to action) and from linear thinking to closed-loop or systems thinking.” |
|  | 19. Towards a new framework for evaluating systemic problem structuring methods – Midgley (2013) 10.1016/j.ejor.2013.01.047 | Paper | “Typical questions addressed by different systemic PSMs [problem structuring methods] include: Whose viewpoints and what aspects of the issue should be included in analysis and decision making, and what should be excluded? (e.g., Ulrich, 1994; Midgley, 2000 ). What are people’s different perspectives on the issue, and what values and assumptions underpin these perspectives? (e.g., Checkland and Scholes, 1990; Checkland and Poulter, 2006 ). What interactions within and across organisational, social and environmental phenomena could produce desirable or undesirable outcomes? (e.g., Vennix, 1996; Maani and Cavana, 2007).”  “Our epistemological argument is that knowledge (or understanding) is always linked to the purposes and values of those producing or using it, and is dependent on the boundary judgements that they make (Churchman, 1970; Ulrich, 1994; Alrøe, 2000; Midgley, 200 ). To claim that knowledge about systemic PSMs [problem structuring methods] (or any other phenomenon for that matter) is universal is to ignore the purposes, values and boundary judgements that make the knowledge relevant and adequate for a particular context.”  “Our evaluation framework is represented in Fig. 1. An evaluation using it is primarily focused on the use of a particular *method* (or set of methods) in a *context* for particular *purposes*, giving rise to *outcomes*.”  “In our approach, when looking at a single case study, there is no pretence that it is possible to evaluate a method independently from the purposes it is put to, its outcomes and the context in which it is applied. Nevertheless, we can still inquire about the relationships between the method, purposes, outcomes and context. Inquiry focused on an intervention can look, for example, at how satisfactorily the method addressed given purposes; what aspects of the context enabled or constrained its application; and whether it gave rise to anticipated or unanticipated outcomes.”  “Within and across ecological, economic, social and organisational systems, there may be important causal pathways, and in particular feedback loops, that can point to systemic enablers of, or constraints on, an intervention (e.g., Forrester ,1969). Bateson (1970) argues that it is important not to ‘cut’ relevant feedback loops, and again this is a good principle to inform boundary critique: when we see interconnections stretching beyond people’s usual understandings of context we can ask whether it is important to widen the boundaries of analysis to account for these.”  “In contrast, outcome criteria refer to whether, in a particular case, the method facilitated the achievement of specific goals (e.g. the production of a plan or the generation of a common vision). The difference between process and outcome criteria can get a little blurred when an explicit goal of an intervention is, for instance, to facilitate participatory engagement. Nevertheless, keeping the distinction explicit helps us avoid potentially major mistakes like focusing so much on process that we fail to notice that people’s purposes for the intervention have not been achieved, or focusing so much on outcomes that we miss negative effects of the process on participants.” |
|  | Systems Thinking, System Dynamics: Managing Change and Complexity – Maani (2007) | Book | *Unavailable.* |
|  | Systems Thinking and Modelling: Understanding Change and Complexity – Maani (2001) | Book | *Unavailable.* |
| **Other key methodological publications especially important to systems approaches in public health** | | **Type** | **Relevant text** |
| 20. Thinking in Systems–A Primer – Meadows (2008) | | Book | “Ever since the Industrial Revolution, Western society has benefited from science, logic, and reductionism over intuition and holism. Psychologically and politically we would much rather assume that the cause of a problem is “out there,” rather than “in here.” It’s almost irresistible to blame something or someone else, to shift responsibility away from ourselves, and to look for the control knob, the product, the pill, the technical fix that will make a problem go away. Serious problems have been solved by focusing on external agents— preventing smallpox, increasing food production, moving large weights and many people rapidly over long distances. Because they are embedded in larger systems, however, some of our “solutions” have created further problems. And some problems, those most rooted in the internal structure of complex systems, the real messes, have refused to go away. Hunger, poverty, environmental degradation, economic instability, unemployment, chronic disease, drug addiction, and war, for example, persist in spite of the analytical ability and technical brilliance that have been directed toward eradicating them. No one deliberately creates those problems, no one wants them to persist, but they persist nonetheless. That is because they are intrinsically systems problems—undesirable behaviors characteristic of the system structures that produce them. They will yield only as we reclaim our intuition, stop casting blame, see the system as the source of its own problems, and find the courage and wisdom to *restructure* it.”  “From this understanding I move into what you and I can do about restructuring the systems we live within. We can learn how to look for leverage points for change.”  “Balancing feedback loops are equilibrating or goal-seeking structures in systems and are both sources of stability and sources of resistance to change.”  “The trick, as with all the behavioral possibilities of complex systems, is to recognize what structures contain which latent behaviors, and what conditions release those behaviors—and, where possible, to arrange the structures and conditions to reduce the probability of destructive behaviors and to encourage the possibility of beneficial ones.”  “This chapter describes some of the reasons why dynamic systems are so often surprising. Alternately, it is a compilation of some of the ways our mental models fail to take into account the complications of the real world—at least those ways that one can see from a systems perspective. It is a warning list. Here is where hidden snags lie. You can’t navigate well in an interconnected, feedback-dominated world unless you take your eyes off short-term events and look for long-term behavior and structure; unless you are aware of false boundaries and bounded rationality; unless you take into account limiting factors, nonlinearities and delays. You are likely to mistreat, misdesign, or misread systems if you don’t respect their properties of resilience, self-organization, and hierarchy.”  “There are no separate systems. The world is a continuum. Where to draw a boundary around a system depends on the purpose of the discussion—the questions we want to ask.”  “When you draw boundaries too narrowly, the system surprises you. For example, if you try to deal with urban traffic problems without thinking about settlement patterns, you build highways, which attract housing developments along their whole length. Those households, in turn, put more cars on the highways, which then become just as clogged as before.”  “It’s a great art to remember that boundaries are of our own making, and that they can and should be reconsidered for each new discussion, problem, or purpose. It’s a challenge to stay creative enough to drop the boundaries that worked for the last problem and to find the most appropriate set of boundaries for the next question. It’s also a necessity, if problems are to be solved well.”  “Change comes first from stepping outside the limited information that can be seen from any single place in the system and getting an overview. From a wider perspective, information flows, goals, incentives, and disincentives can be restructured so that separate, bounded, rational actions do add up to results that everyone desires.”  “To paraphrase a common prayer: God grant us the serenity to exercise our bounded rationality freely in the systems that are structured appropriately, the courage to restructure the systems that aren’t, and the wisdom to know the difference!”  “Understanding archetypal problem-generating structures is not enough. Putting up with them is impossible. They need to be changed. The destruction they cause is often blamed on particular actors or events, although it is actually a consequence of system structure. Blaming, disciplining, firing, twisting policy levers harder, hoping for a more favorable sequence of driving events, tinkering at the margins—these standard responses will not fix structural problems. That is why I call these archetypes “traps.” But system traps can be escaped—by recognizing them in advance and not getting caught in them, or by altering the structure—by reformulating goals, by weakening, strengthening, or altering feedback loops, by adding new feedback loops. That is why I call these archetypes not just traps, but opportunities.”  “Policy Resistance—Fixes that Fail. As we saw in Chapter Two, the primary symptom of a balancing feedback loop structure is that not much changes, despite outside forces pushing the system. Balancing loops stabilize systems; behavior patterns persist. This is a great structure if you are trying to maintain your body temperature at 37°C (98.6°F), but some behavior patterns that persist over long periods of time are undesirable. Despite efforts to invent technological or policy “fixes,” the system seems to be intractably stuck, producing the same behavior every year. This is the systemic trap of “fixes that fail” or “policy resistance.” You see this when farm programs try year after year to reduce gluts, but there is still overproduction. There are wars on drugs, after which drugs are as prevalent as ever. There is little evidence that investment tax credits and many other policies designed to stimulate investment when the market is not rewarding investment actually work. No single policy yet has been able to bring down health care costs in the United States. Decades of “job creation” have not managed to keep unemployment permanently low. You probably can name a dozen other areas in which energetic efforts consistently produce non-results. Policy resistance comes from the bounded rationalities of the actors in a system, each with his or her (or “its” in the case of an institution) own goals. Each actor monitors the state of the system with regard to some important variable—income or prices or housing or drugs or investment— and compares that state with his, her, or its goal. If there is a discrepancy, each actor does something to correct the situation. Usually the greater the discrepancy between the goal and the actual situation, the more emphatic the action will be. Such resistance to change arises when goals of subsystems are different from and inconsistent with each other. Picture a single-system stock—drug supply on the city streets, for example—with various actors trying to pull that stock in different directions. Addicts want to keep it high, enforcement agencies want to keep it low, pushers want to keep it right in the middle so prices don’t get either too high or too low. The average citizen really just wants to be safe from robberies by addicts trying to get money to buy drugs. All the actors work hard to achieve their different goals.”  “THE TRAP: POLICY RESISTANCE When various actors try to pull a system stock toward various goals, the result can be policy resistance. Any new policy, especially if it’s effective, just pulls the stock farther from the goals of other actors and produces additional resistance, with a result that no one likes, but that everyone expends considerable eff ort in maintaining. THE WAY OUT Let go. Bring in all the actors and use the energy formerly expended on resistance to seek out mutually satisfactory ways for all goals to be realized—or redefinitions of larger and more important goals that everyone can pull toward together.”  “The only other graceful way out of the escalation system is to negotiate a disarmament. That’s a structural change, an exercise in system design. It creates a new set of balancing controlling loops to keep the competition in bounds (parental pressure to stop the kids’ fight; regulations on the size and placement of advertisements; peace-keeping troops in violence-prone areas). Disarmament agreements in escalation systems are not usually easy to get, and are never very pleasing to the parties involved, but they are much better than staying in the race.”  “Again, the best way out of this trap is to avoid getting in. Beware of symptom-relieving or signal-denying policies or practices that don’t really address the problem. Take the focus off short-term relief and put it on long-term restructuring.”  “So, how do we change the structure of systems to produce more of what we want and less of that which is undesirable?”  “Counterintuitive—that’s Forrester’s word to describe complex systems. Leverage points frequently are not intuitive. Or if they are, we too often use them backward, systematically worsening whatever problems we are trying to solve.”  “6. Information Flows—The structure of who does and does not have access to information. In Chapter Four, we examined the story of the electric meter in a Dutch housing development—in some of the houses the meter was installed in the basement; in others it was installed in the front hall. With no other differences in the houses, electricity consumption was 30 percent lower in the houses where the meter was in the highly visible location in the front hall. I love that story because it’s an example of a high leverage point in the information structure of the system. It’s not a parameter adjustment, not a strengthening or weakening of an existing feedback loop. It’s a new loop, delivering feedback to a place where it wasn’t going before.”  “As we try to imagine restructured rules and what our behavior would be under them, we come to understand the power of rules. They are high leverage points. Power over the rules is real power. That’s why lobbyists congregate when Congress writes laws, and why the Supreme Court, which interprets and delineates the Constitution—the rules for writing the rules—has even more power than Congress. If you want to understand the deepest malfunctions of systems, pay attention to the rules and to who has power over them.”  “4. Self-Organization—The power to add, change, or evolve system structure. The most stunning thing living systems and some social systems can do is to change themselves utterly by creating whole new structures and behaviors. In biological systems that power is called evolution. In human economies it’s called technical advance or social revolution. In systems lingo it’s called self-organization. Self-organization means changing any aspect of a system lower on this list—adding completely new physical structures, such as brains or wings or computers—adding new balancing or reinforcing loops, or new rules.”  “You don’t have to put forth your mental model with diagrams and equations, although doing so is a good practice. You can do it with words or lists or pictures or arrows showing what you think is connected to what. The more you do that, in any form, the clearer your thinking will become, the faster you will admit your uncertainties and correct your mistakes, and the more flexible you will learn to be. Mental flexibility—the willingness to redraw boundaries, to notice that a system has shifted into a new mode, to see how to redesign structure—is a necessity when you live in a world of flexible systems.” |
| 21. Leverage points: Places to intervene in a system – Meadows (1999) | | Paper | “Counterintuitive. That’s Forrester’s word to describe complex systems. Leverage points are not intuitive. Or if they are, we intuitively use them backward, systematically worsening whatever problems we are trying to solve.”  “The plumbing structure, the stocks and flows and their physical arrangement, can have an enormous effect on how the system operates. When the Hungarian road system was laid out so all traffic from one side of the nation to the other has to pass through central Budapest, that determined a lot about air pollution and commuting delays that are not easily fixed by pollution control devices, traffic lights, or speed limits. The only way to fix a system that is laid out wrong is to rebuild it, if you can.”  “We systems-heads love that story because it’s an example of a high leverage point in the information structure of the system. It’s not a parameter adjustment, not a strengthening or weakening of an existing loop. It’s a *new loop*, delivering information toa place where it wasn’t going before and therefore causing people to behave differently.”  “As we try to imagine restructured rules like these and what our behavior would be under them, we come to understand the power of rules.”  “The most stunning thing living systems and social systems can do is to change themselves utterly by creating whole new structures and behaviors.”  “Self-organization means changing any aspect of a system lower on this list: adding completely new physical structures, such as brains or wings or computers; adding new negative or positive loops; making new rules.” |
| 22. Systems science and obesity policy: A novel framework for analyzing and rethinking population-level planning – Johnston (2014) 10.2105/AJPH.2014.301884 | | Paper | “Although the Foresight map helps to focus dialogue on the complex nature of obesity, it does not immediately lead to discussion of solutions appropriate for this complex problem. We recently developed a systems science framework that may be a useful and accessible means of operationalizing systems thinking toward solutions. The intervention-level framework (ILF) was adapted from Donella Meadows’^15^ list of 12 places to intervene in complex systems. Meadows, a pioneering environmental scientist, spent decades analyzing the complexities of economic growth and environmental sustainability, and she grew frustrated with the unintended consequences that resulted when simple solutions were applied to complex problems.”  “System structure: Interconnections between system elements and subsystems – Actions at this level will shift the system structure by changing system linkages or incorporating novel elements”  “Activities coded as system structure included efforts to build collaborations across subsystems, thereby forging connections between new parties and expanding the boundaries of subsystems.”  “Sterman^40^ noted that a failure to focus on feedback in policy design has critical consequences, prompting us to reconsider the role of feedback and delays in future planning.”  “Activities aimed at the level of system structure have the potential to shift both the physical components of a system and the flow of information among its players.”  “Cross-sector collaboration across subsystems also has the potential to network like-minded social movements and synergistically increase their impact^42^ while potentially addressing the system trap of policy resistance, which Meadows^15^ suggested is partly attributable to the competing interests of system actors.”  “Theorists have argued that changes in the obesity system will ultimately be grounded in shifting social norms and cultures^6,43^; improving the dissemination of knowledge and innovation throughout system networks through activities targeting the system structure level may contribute to this shift.” |
